# The meaning of touch: Relational and individual variables shape emotions and intentions associated with imagined social touch

**DOI:** 10.1002/ejsp.3076

**Published:** 2024-06-04

**Authors:** Charlotte Krahé, Aikaterini Fotopoulou, Claudia Hammond, Michael J. Banissy, Athanasios Koukoutsakis, Paul M. Jenkinson

**Affiliations:** 1 School of Psychology, Faculty of Health, Liverpool John Moores University, Liverpool, UK; 2 Research Department of Clinical, Educational and Health Psychology, University College London, London, UK; 3 Department of Psychology, University of Sussex, Falmer, UK; 4 Department of Psychology, Goldsmiths, University of London, London, UK; 5 School of Psychological Science, University of Bristol, Bristol, UK; 6 Faculty of Psychology, Counselling and Psychotherapy, The Cairnmillar Institute, Melbourne, Australia

**Keywords:** affective touch, attachment style, communication, emotion, intention, social touch

## Abstract

Touch is a key channel for conveying meaning in social interactions. The affective quality of touch and its effects on well-being are shaped by relational context (relationship between touch giver vs. recipient) and person variables (e.g. adult attachment style). Yet, such effects have not been explored in relation to the *meaning* ascribed to touch. We used data from the Touch Test, the world’s largest touch survey, which included questions on the degree to which people felt and related specific emotions and intentions to imagined gentle stroking touch and hugs. In *N* = 23,428, we examined how relational context (imagined source of touch) and person variables (gender, recalled positive childhood touch and adult attachment style) were associated with positive (e.g. love, desire, support) and negative (e.g. fear, anger, warning) emotions and intentions related to imagined touch. Love, desire and support were endorsed more when participants had had their partner (vs. someone else) in mind, and women (vs. men) gave lower ratings for desire overall. Gentle stroking touch was most linked with arousal when participants had had their partner in mind. Further, more positive childhood touch and secure and anxious attachment scores were associated with more positive emotions and intentions, while the opposite was found for avoidant attachment scores. Lastly, positive childhood touch and higher anxious attachment scores were related to greater discrimination between distinct emotion and intention categories, while higher attachment avoidance was associated with reduced discriminability. Thus, contextual and person variables matter in shaping the meaning of social touch.

## 1 Introduction

Our sense of touch is integral for exploring and communicating with the world around us. We touch surfaces and objects to understand their properties, move them, and use them. We touch other people, and are touched by other people, to convey meaning (e.g. love or support; Hertenstein et al., 2006; Heslin et al., 1983; Kirsch et al., 2018; McIntyre et al., 2022; Nguyen et al., 1975, 1976) and to influence other people, such as affecting their emotions (e.g. soothe and buffer their stress; Van Puyvelde et al., 2019; von Mohr et al., 2017) or behaviours (e.g. the famous example that touch increases restaurant tipping; Crusco & Wetzel, 1984). These two functions of social touch have been considered in ‘signal’ and nonverbal ‘effecting’ models, respectively (Schirmer et al., 2022), though the two functions are arguably linked, with the meaning (i.e. signal) conveyed by touch influencing its effects (Sailer & Leknes, 2022). Crucially, both the *affective quality* of touch (e.g. its pleasantness) and *touch effects* are shaped by relational context and person variables, such as personality traits. Regarding context, for example, the soothing effects of stroking touch in 9-month-old infants were reversed when infants believed they were being stroked by a stranger rather than their parent (Aguirre et al., 2019). In adulthood, the perceived pleasantness of such stroking touch is modulated by how much touch people are generally exposed to (Sailer & Ackerley, 2019) and personality traits, such as their mental representations of close relationships (adult attachment styles; Krahé et al., 2018; Spitoni et al., 2020). How relational context and person variables shape the imagined *meaning* that is ascribed to social touch was explored by a handful of studies more than four decades ago showing that gender and relational closeness may affect the meaning of touch (Heslin et al., 1983; Nguyen et al., 1975), but the topic has received less attention since, with only a few exceptions (e.g., Price et al., 2022). Therefore, in the present study, we used data from the world’s largest survey on touch to date (the Touch Test) to examine how relational context and person variables shape the meaning of imagined social touch two decades into the 21st century.

The Touch Test focused on prosocial touch, specifically hugs and gentle caressing touch, in the tradition of viewing such forms of touch as critical for positively regulating others’ affective states. From birth, our caregivers touch us to help us reduce negative affective states, such as pain, hunger or feeling cold, by stroking or holding, feeding or dressing us (Fotopoulou & Tsakiris, 2017). These early touch experiences set the stage for affect regulation through social touch across the lifespan (see Fotopoulou et al., 2022, for a theoretical review). A wealth of evidence supports the idea that social touch reduces negative affective states such as emotional pain (von Mohr et al., 2017), physical pain (von Mohr et al., 2018), and stress (Morrison, 2016), even when touch is imagined rather than directly experienced (Jakubiak & Feeney, 2016a). Furthermore, social touch also exerts positive effects on well-being (Debrot et al., 2020; Field, 2019; Jakubiak & Feeney, 2017), in part through touch promoting the formation and maintenance of close social bonds (Bendas & Croy, 2021) and the perception of those bonds (Jakubiak & Feeney, 2016). For example, Jakubiak and Feeney (2016) found that receiving touch (holding hands, arms around body) from the romantic partner was associated with greater self-reported state attachment security, including feeling safe, comforted, and loved. As well as hand-holding and hugs, a specific type of slow, gentle caressing touch seems especially important in facilitating prosocial approach behaviour (Pawling et al., 2017) and strengthening social bonds and intimacy between people. Slow, gentle stroking at speeds of 1–10 cm/s optimally activates a class of unmyelinated C tactile (CT fibres) in the skin, and activation of CT fibres is positively correlated with perceived pleasantness (Löken et al., 2009). Thus, this type of social touch is often termed ‘affective touch’ as – compared to faster stroking touch at ‘non-CT-optimal’ speeds – it has a positive hedonic valency: It generally feels pleasant (Löken et al., 2009).

However, contextual and individual factors shape the perceived pleasantness of touch. In adults, the strength of the emotional bond with the person providing touch is positively related with touch pleasantness and touch permissibility (Heslin et al., 1983; Suvilehto et al., 2019). In close relational contexts, such as romantic relationships, slow gentle stroking touch is perceived as pleasant and erotically arousing (Bendas et al., 2017; Nguyen et al., 1975; Panagiotopoulou et al., 2018), but this appears to depend on gender, with women rating slow, gentle touch as more erotic than men (Bendas et al., 2017). Examining gender differences and degree of familiarity, women and men (though surveyed 40 years ago) differed in terms of perceived pleasantness when touch was imagined from an opposite-sex friend versus a stranger, with men rating touch from known and unknown women as equally pleasant, while women rated touch from a male stranger as significantly less pleasant than touch from a male friend (Heslin et al., 1983).

Personality traits are also related to differences in the perceived affective quality of touch. In particular, attachment styles, including mental representations regarding the availability of close others to one’s needs, influence the perceived pleasantness of slow, gentle touch (e.g. Krahé et al., 2018) and its effect on negative affective states, such as pain (Krahé et al., 2016). Attachment styles develop through early experiences with caregivers, which critically include early touch experiences, such as caregivers signalling affection and closeness through touch and using touch to fulfil children’s basic needs (Fotopoulou et al., 2022). Prosocial and especially affective touch is key in maintaining proximity to caregivers and forming secure attachment bonds (e.g. Bendas & Croy, 2021), and (cross-sectionally) attachment styles are linked to affective touch experiences (Beltrán et al., 2020). Importantly, past touch experiences and attachment styles appear key in the ability to discriminate between different types of touch in terms of their pleasantness. For example, fewer overall experiences of being touched are associated with a poorer ability to discriminate between CT-optimal and non-CT-optimal touch on the basis of pleasantness (Sailer & Ackerley, 2019). This reduced discriminability is also seen in individuals with a more insecure attachment style, and especially at higher levels of attachment anxiety (Krahé et al., 2018). Distinctiveness of stroking velocities – in terms of perceived pleasantness – is thus shaped by individual differences. However, it is unclear whether the distinctiveness of different emotions and intentions associated with touch also varies by touch experiences and attachment styles. Overwhelmingly, studies have focused on perceived pleasantness of touch as the outcome, and have not examined how the meaning ascribed to touch, and the discriminability between different possible meanings, might be affected by relational context (relationship with toucher) and person variables (gender, touch history, and attachment styles).

Touch serves as a non-verbal channel of communicating meaning. Focusing on conveying emotions, Hertenstein et al. (2006) demonstrated that individuals can decode discrete prosocial emotions from different types of touch; for example, love was reliably decoded from gentle stroking, either experienced or observed (see also Nguyen et al., 1975). McIntyre et al. (2022) further showed that messages (construed more broadly than emotions, and including attention, love, happiness, calming, sadness, and gratitude) could be identified by receivers within a close-relationship context. Further, when core features of the touches conveying these messages were extracted to construct ‘standardised’ touch profiles, participants could also decode the intended messages when standardised touches were provided by strangers. Messages of love and calming involved slow, stroking movements as opposed to, for example, tapping movements to indicate happiness or attention (McIntyre et al., 2022). Focusing specifically on emotions and intentions conveyed by slow, gentle (CT-optimal) touch versus faster non-CT-optimal speeds, Kirsch et al. (2018) found that stroking at CT-optimal velocities was interpreted as arousal/desire (emotion) and social support (intention). Faster touch, on the other hand, was perceived to convey joy/fear (emotions) and warning (intention). Importantly, however, such studies had relatively small sample sizes, and did not directly investigate the influence of different relational contexts and person variables on self-reported meaning from touch; that is, how touch is interpreted or ‘read’ depending on contextual and personal characteristics.

While most studies have examined touch that is directly experienced, individuals can think about what touch means without receiving sensory input, as demonstrated by early studies on this topic that asked people to self-report meanings they ascribed to touch to various body parts from different persons (Heslin et al., 1983; Nguyen et al., 1975). Imagined touch seems to convey and elicit some of the prosocial effects of experienced touch. For example, imagining affective touch induces feelings of pleasantness (Panagiotopoulou et al., 2018) and activates neural regions (anterior insula) involved in interpreting the affective meaning of the touch (Lucas et al., 2014). Imagined touch can also influence positive cognitions (e.g. around state attachment security; Jakubiak & Feeney, 2016), can buffer against stress (Jakubiak & Feeney, 2016a) and can reveal individual differences in, for example, the enjoyment or perceived importance of touch (Carmichael et al., 2021). Imagined touch further affords advantages compared to actual touch: Not everyone has opportunities for actual touch. For example, people living far from their families while travelling for work, living in residential care or hospitalised in isolation for medical reasons (such as during the Covid-19 pandemic) may experience touch deprivation. Such deprivation has been associated with negative consequences. In the early stages of the Covid-19 pandemic, touch deprivation was related to increased anxiety and feelings of loneliness (von Mohr et al., 2021), along with indications of broader mental health concerns (Field et al., 2020). Consequently, measures and practical solutions to mitigate social touch deprivation, and related anxiety and loneliness, have gained significant attention in both scientific and policy domains. Studying the prosocial effects of imagined touch may have practical utility in developing such solutions in future studies. Moreover, several studies have shown that adding touch to various digital, as well as robotic, forms of communication can have beneficial effects for both the individual and communication between individuals (Saramandi et al., 2023). However, these studies have also made it clear that the meaning of the touch is dependant as much on what people sense as it is on what they imagine. Hence, studying how people imagine touch communication has the potential to inform digital touch in unique ways. Furthermore, imagined rather than experienced touch may be particularly well-suited for illuminating variations in touch meaning by individual differences, as imagining touch likely draws on episodic memories, with content and specificity of episodic details known to vary by, for example, attachment style (Cao et al., 2018). Studying imagined touch without sensory stimulation in a large sample has the potential of informing us of how individual differences might shape touch meaning. In the present study, we thus investigated which emotions and intentions people associate with imagined touch, and which individual and relational factors moderate these associations.

We accessed data from the Touch Test (Bowling et al., 2023 AU), in which nearly 40,000 participants were asked about different aspects of touch, including which emotion and intentions they would associate with two types of prosocial touch, namely hugs and slow, gentle touch. Participants imagined these types of touch and indicated who they had in mind when responding to questions (i.e. they were able to choose touch source and were not instructed to imagine touch from specific sources). Although testing imagined touch with a self-report methodology has limitations (see Section 4), the large sample of our survey, derived from the general UK population, allowed us to test a series of pre-registered hypotheses (https://osf.io/gvjqz) examining the influence of attachment style, touch history, and gender on touch meaning. First, to replicate existing studies (e.g. Hertenstein et al., 2006; Kirsch et al., 2018; McIntyre et al., 2022; Nguyen et al., 1975), we tested the hypothesis that people would generally interpret slow, gentle caressing touch and hugs as evoking and communicating positive (rather than negative) emotions and social intentions (H1). Next, we examined how interpretation of touch would vary by relational context, with the caveat that context was chosen by participants. Based on the wealth of literature regarding the links between social touch and close emotional bonds, we tested the hypothesis that people would interpret slow, gentle touch and hugs as eliciting and indicating more positive emotions and intentions (specifically, desire, love, and social support) when participants chose to respond by thinking of their partner compared to by thinking of someone else (H2). We also explored gender differences, hypothesising that women would be more likely than men to report love, desire, and social support from their partner (vs. others; Bendas et al., 2017), whereas men were expected to report more desire than women regardless of touch source (Heslin et al., 1983; H2.1). Next, following previous work (Bendas et al., 2017; Panagiotopoulou et al., 2018), we focused on erotic arousal, testing the hypothesis that people would interpret slow, gentle, caressing touch as more erotic (arousal/lust/desire emotions and intentions) than a hug, particularly when they chose to have their partner in mind (H3). Finally, we examined individual differences in attachment style and touch history (amount of positive parental touch in childhood) to test the hypothesis that less secure attachment (higher scores on attachment anxiety and avoidance dimensions) and less touch during childhood would be associated with rating slow, gentle caressing touch and hugs as eliciting and conveying less positive (H4.1) and less distinct (H4.2) emotions and intentions.

## 2 Methods

### 2.1 Design and procedure

The study employed a cross-sectional survey design. Data were obtained online through the ‘Touch Test’. The Touch Test survey was created by Goldsmiths (University of London) and University College London as academic partners, and organised by the Wellcome Collection in collaboration with the British Broadcasting Corporation (BBC). The survey was accessed via a purpose-built online platform (www.touchtest.org). It was launched by BBC Radio 4 on 21 January 2020, and was widely advertised to the general public in the United Kingdom through radio broadcasts and social media by BBC radio and the Wellcome Collection. It remained open to the public for completion until 30 March 2020. On accessing the survey, participants were presented with an information sheet and provided informed consent before proceeding to the survey. The survey had two parts, and participants could choose to complete one or both parts. The ratings and measures included in this paper were drawn from both parts of the questionnaire; thus, only participants who completed both parts were included. Questionnaires in each part were presented in a random order, and the full survey was expected to take 30–45 min to complete. Participants could interrupt and return to the survey as many times as they wanted within 7 days of starting. However, most participants (89.76%) completed the survey in 1 day. The study was approved by the Research Ethics and Integrity Sub-Committee, Goldsmiths, University of London.

**Figure 1:**
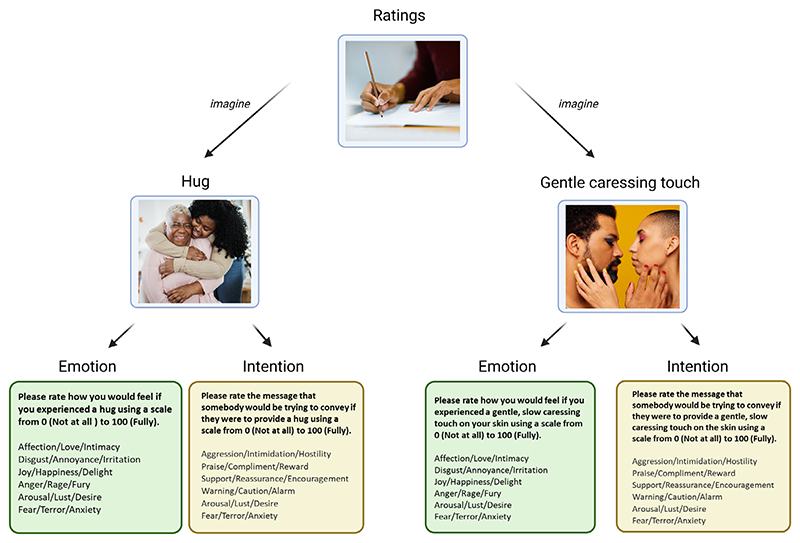
Breakdown of touch ratings for hugs and gentle caressing touch. Participants gave six emotion and six intention ratings separately for hugs and gentle caressing touch, resulting in 24 ratings. Afterwards, they indicated who they had had in mind when making their ratings.

In this paper, we focused on specific parts of the larger survey relevant to testing our hypotheses (https://osf.io/gvjqz). We report only data from questions asking participants to imagine slow, gentle touch and hugs, and rate different emotion and intention terms as well as who they had in mind when rating these, and relevant demographic variables and self-report measures of developmental touch history and attachment style. The emotion and intention terms (e.g. love, fear, support, warning) were taken from Kirsch et al. (2018) and are presented in Figure 1 and in Section 2.3.2. Briefly, participants rated six emotions and six intentions in relation to (a) imagined gentle, stroking touch and (b) hugs, yielding 24 ratings in total. Outcomes of interest were mean ratings for emotions and intentions (see Section 2.3.2). Additionally, we computed distinctiveness scores (see Section 3) to examine effects of individual differences on the discriminability of different emotion and intention terms. Predictors, depending on hypothesis, were touch source (partner vs. other), touch type (gentle touch vs. hugs), touch valence (negative vs. positive, categorised based on the emotion and intention terms), specific emotion or intention categories (see below), gender (identifying as men or women), attachment anxiety and avoidance (continuous scores), and developmental touch history (continuous score). We also controlled for covariates linked theoretically to touch ratings, such as general touch experiences and attitudes (see next).

### 2.2 Participants

Members of the general population were invited to take part in the world’s largest online survey on touch. *N* = 39,254 participants completed at least part of the survey. The open nature of this survey meant that we received responses from an extremely heterogenous sample. As pre-registered, we only included participants self-identifying as men or women, living in the United Kingdom, and aged 19 and above (because in Tanzer et al., 2022, participants who reported being aged 18 were excluded due to a disproportionately high number of respondents in this age category, which can indicate that individuals under the age of 18 completed the survey and selected this age). This left *N* = 23,475 predominantly white (95.7%) participants with a mean age (*SD*) of 57.05 years (13.92; range 19–94) of whom 25.2% self-identified as men and 74.8% as women (see Table 1 for full demographic information). Almost all participants completed the survey before the first UK Covid-19 lockdown began on 23 March 2020, but we nevertheless controlled for when the survey was completed in analyses to account for any effects of the onset of the pandemic. Just fewer than half of participants indicated that the last time somebody had touched them ‘intentionally, not including formal gestures such as handshakes in meetings?’ was in the last day or less (45.9%). We controlled for last time people were touched in our analyses. As pre-registered, participants who completed fewer than 80% of items on the various measures were excluded from analyses (see Section 2.4). Therefore, there was a slightly different *N* in each analysis, and the total *N* is reported for each analysis.

### 2.3 Materials and measures

#### 2.3.1 Demographic information

We accessed information on age, gender, sexuality, ethnicity, and date of survey completion (to control for the possible influence of the Covid-19 restrictions).

#### 2.3.2 Touch rating outcome

Participants were asked to rate emotions they would feel and intentions which they felt were conveyed by gentle, slow caressing touch and hugs (see Figure 1). Specifically, for emotions, they were asked (separately for gentle touch and hug, but presented together here for parsimony), ‘Please rate how you would feel if you experienced a gentle, slow caressing touch / hug on your skin using a scale from 0 (not at all) to 100 (fully)’ and for intentions, ‘Please rate the message that somebody would be trying to convey if they were to provide a gentle, slow caressing touch on the skin / hug using a scale from 0 (not at all) to 100 (fully)’.

Then, six emotion categories/six intention categories were provided based on previous work (see Kirsch et al., 2018). Each emotion category contained three semantically-related words that described the represented emotion. Positive emotions included (1) affection/love/intimacy (love as the overarching category with linked concepts), (2) joy/happiness/delight (happiness) and (3) arousal/lust/desire (desire). Negative emotions included (1) disgust/annoyance/irritation (annoyance as the umbrella concept), (2) anger/rage/fury (anger) and (3) fear/terror/anxiety (fear). For intentions, each category also contained three semantically-related words. Positive intentions included (1) support/reassurance/encouragement (support as overarching concept), (2) praise/compliment/reward (praise) and (3) arousal/lust/desire (desire). Negative intentions included (1) aggression/intimidation/hostility (aggression), (2) warning/caution/alarm (warning) and (3) fear/terror/anxiety (fear). Of note, fear as an intention referred to communicating fear and anxiety, rather than intending to induce fear (e.g. to threaten). We did not create averages for emotion or intention ratings; instead, all six emotion categories/six intention categories were concurrently entered into each multivariate multilevel model as dependent variables (see Section 2.4).

#### 2.3.3 Relational context predictor

After providing the ratings, participants were asked, ‘Who did you have in mind when you were answering the last set of questions?’ and were shown the options of ‘a friend’, ‘a partner’, ‘a family member’, ‘a stranger’, ‘no one in particular’, or ‘someone else [specify in free text]’. Not everyone answered this question; of those who did (*N* = 16,193), most participants indicated having had their partner in mind (*N* = 8576), followed by a friend (*N* = 2630), no one in particular (*N* = 2586), a family member (*N* = 1150), someone else (*N* = 963) and a stranger (*N* = 288). As relational context hypotheses pertained to romantic partner versus someone else, we created a binary predictor variable for touch source (partner vs. other) from these six categories but also explored specific comparisons (see Section 2.4).

#### 2.3.4 Individual differences predictors

*Adult attachment style*: Adult attachment style was measured using the 12-item short form of the Experiences in Close Relationships Questionnaire (Lafontaine et al., 2016), yielding scores on adult attachment avoidance (captured by six items, such as, ‘I don’t feel comfortable opening up to romantic partners’) and anxiety (six items, such as, ‘I worry about being abandoned’) dimensions. Items were rated from 1 (strongly disagree) to 7 (strongly agree), with higher average scores denoting higher attachment anxiety (Cronbach’s *α* = 0.86) and avoidance (*α* = 0.86), respectively. These dimensions were entered into the models as continuous predictors together with their interaction term, as pre-registered. Where interactions between continuous predictors were significant, we drew on Aiken and West’s (1991) recommendations for probing and plotting interactions between continuous variables. Here, too, participants were not placed into categories, but rather we estimated marginal means for the attachment anxiety by attachment avoidance interaction at combinations of −1*SD* and +1*SD* of these two continuous attachment dimensions. Specifically, we conceptualised attachment style as secure (operationalised as 1*SD* below the sample mean on both attachment anxiety and avoidance) or insecure, with the latter divided into different types of insecure attachment (see Bartholomew & Horowitz, 1991) in the following ways: We interpreted and plotted scores of +1*SD* anxiety/−1*SD* avoidance as anxious attachment (preoccupied in Bartholomew & Horowitz, 1991, but we chose the terms used in the ECR-S), −1*SD* anxiety/+1*SD* avoidance scores as avoidant attachment (dismissing in Bartholomew & Horowitz, 1991) and +1*SD* scores on both dimensions as fearful attachment. We tested whether marginal means for these combinations of different *SD*s above and below the means (i.e. the attachment styles) differed significantly from each other and plotted these data.

*Positive childhood touch*: To assess how much positive touch participants had received in childhood, we created a composite average score of two items from the Childhood Touch subscale of the Touch Experiences and Attitudes Questionnaire (TEAQ; Trotter et al., 2018). Only these two items were included in the survey from the longer subscale; the items included were those that loaded most strongly on the Childhood Touch subscale, namely, ‘My parents were not very physically affectionate towards me during my childhood’ (item 9, reversed) and ‘As a child, my parents would tuck me up in bed every night and give me a hug and a kiss goodnight’ (item 22). Participants rated how much they agreed with each item on a 5-point Likert scale ranging from ‘disagree strongly’ (1) to ‘agree strongly’ (5). Items were averaged, and higher scores denoted a more positive developmental touch history. Cronbach’s alpha was *α* = 0.75.^1^

#### 2.3.5 Covariates

We controlled for variables that we considered to possibly be linked to ratings of emotions and intentions regarding imagined hugs and gentle touch.

*Time when survey was completed*: To account for any effects of the onset of Covid-19 pandemic-related social restrictions in the United Kingdom (first national lockdown commenced on 23 March 2020), we included, as a continuous variable, the number of weeks since the beginning of 2020 that had elapsed at the point of survey completion for each participant.

*Attitudes towards intimate touch*: Given that the imagined touch related to hugs and slow, gentle caresses, we assessed participants’ general attitudes to intimate touch. *Attitude to Intimate Touch* is one of six subscales of the TEAQ. For this subscale, the two highest loading items were included here, namely, ‘I like to stroke the skin of someone I know intimately’ and, ‘I enjoy the feeling of my skin against someone else’s if I know them intimately’. Items were scored using the Likert scale outlined above (see Positive childhood touch), averaged, and higher scores denoted more positive attitudes to intimate touch (Cronbach’s *α* = 0.76).^1^

*Recent touch experiences*: As differences in the perceived affective quality of touch have been reported to vary as a function of touch exposure (Sailer & Ackerley, 2019), we asked participants about their recent touch exposure. Participants were asked, ‘When was the last time that somebody touched you intentionally, not including formal gestures such as handshakes in meetings?’ and indicated whether this last touch had occurred ‘In the last hour’, ‘In the last day or less’, ‘In the last week or less’, ‘In the last month or less’, ‘Over a month ago’, or ‘Over a year ago’. This item was included as a fixed-effect categorical covariate (six levels) in analyses.

*Ability to empathise*: Decoding emotions and intentions from imagined touch might be shaped by a person’s general ability to understand somebody else’s emotional state. Therefore, we controlled for ability to empathise as measured by the 10-item Empathy Quotient-Short Form (EQ-10; Wakabayashi et al., 2006). Participants rated their agreement with statements such as, ‘I really enjoy caring for other people’, with higher scores denoting greater self-reported empathy (Cronbach’s *α* = 0.77).

*Perceived ability to notice bodily signals*: Slow, gentle, caressing touch has been conceptualised as an interoceptive modality (McGlone et al., 2014) and people differ in their ability to sense interoceptive signals. Here, we controlled for interoceptive sensibility, that is, self-reported accuracy in sensing bodily signals, by including a single item from the Interoceptive Accuracy Scale (Murphy, 2018) focusing on how accurate people think they are at discriminating between affectionate and non-affectionate touch: ‘I can always accurately perceive when someone is touching me affectionately rather than non-affectionately’. Responses were captured on a 5-point scale from ‘Strongly Agree’ (5) to ‘Strongly Disagree’ (1). This single item was included as a fixed effect covariate in our analyses.

### 2.4 Plan of analysis

The analysis plan for this study was pre-registered on the OSF (https://osf.io/gvjqz). The dataset used in the analyses can be accessed here: https://osf.io/qt53j/. An example data excerpt for one participant is included in Supporting Information Table 1 to demonstrate how the data were laid out. All analyses were carried out in Stata 16 (StataCorp, 2019), and effect sizes were calculated in R (version 4.2.1). Any changes to the plan of analysis and additional exploratory analyses that were not part of the pre-registration are outlined below and in Section 3. Given the overall large sample size of the survey, some of our analyses were assumed to have very high statistical power, and even very small effects would be identified as significant (*p* < .05). However, the modular nature of the survey and expected variability in the sample size of each analysis (due to incomplete surveys and missing data; see next), meant that some sub-analyses would have a much smaller sample and lower statistical power than others. Therefore, we did not apply a standard cut-off for a meaningful effect size of interest but instead reported the effect size and statistical significance of our analyses so that the basis of our interpretations would be transparent. We used *p* = 0.05 and Bonferroni correction (where applicable and specified) to control the Familywise Error Rate (i.e. when conducting multiple post hoc comparisons and planned contrasts). Assumptions and transformations are outlined on the OSF link and in Supporting Information.

Where individual cases possessed demographic characteristics that are extremely rare and under-represented (e.g. <1% of respondents), we excluded these individuals from our analyses. Thus, we included only men and women in our analysis on gender effects, and limited our analyses to UK residents (see also Section 2.2). Regarding missing data, participants needed to have completed at least 80% of each scale or subscale (e.g. subscales of the ECR-12) for that data to be included in an analysis (see pre-registration). Where fewer than 80% of items were completed, data were excluded. Subscales were treated individually; a participant could be excluded from analysis for one subscale but still be included in analyses for other subscales on that scale, where data were sufficient. If analysis focused on a single item/question from the survey, we included all participants who completed the question.

We ran stepwise multivariate multilevel modelling (MMLM) to examine our predicted effects. Emotions and intentions were examined in separate MMLMs. All six emotion categories/six intention categories were concurrently entered into each MMLM as dependent variables. In each of these analyses, last time participants were touched, ability to empathise, attitudes to intimate touch, week of the year in which the survey was completed (Covid-19 control), and interoceptive sensibility were included as (fixed effect) covariates. All relevant independent variables, such as touch type (hug vs. gentle caressing touch), touch source (partner vs. somebody else), attachment style (anxiety, avoidance, and their interaction) and positive childhood touch were entered as fixed effects of interest. In analyses in which we did not include a certain independent variable, all relevant data rows were included (e.g. when we ran analyses irrespective of touch type, data for both touch types were included). Gender (men vs. women) was included as a fixed effect as described in specific hypotheses. The intercept of Participant ID was included as a random effect. We deviated from our pre-registered analysis to include recent touch experiences (last time touched) as an additional fixed covariate. Further, we did not include demographic factors as random effects as we included only participants living in the United Kingdom.

Within this stepwise analysis, from each model to the next, we added independent variables. Rather than using ANOVA to decide whether including independent variables improved the explanatory power of the model, we used the Akaike information criterion (AIC) to decide whether to retain the more complex models; lower AIC indicates a better-fitting model. The analysis took the following sequential steps: (a) we used the random effect as the baseline model, (b) we evaluated the effect of any covariates with respect to the baseline, (c) we evaluated the effects of independent variables and their interactions with respect to the previous steps, and (d) where two- and three-way interactions were identified, we performed planned contrasts to identify the effects driving the interactions of interest. In exploratory analyses, we also conducted the same analyses with the same steps for only the people who stated that they had thought of a current partner.

## 3 Results

**Figure 2:**
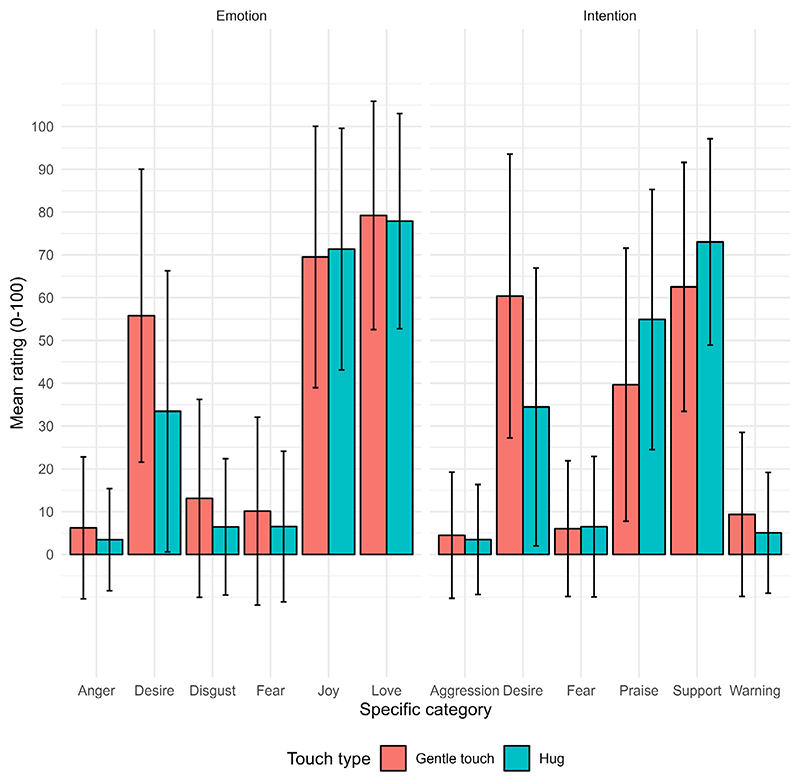
Mean ratings for each specific emotion (left panel) and intention (right panel) category by type of touch (gentle touch; hugs). Error bars denote ±1*SD*.

Descriptive statistics for all ratings are presented in Figure 2 (values in Supporting Information Table 2). Without considering the role of relational context and personal variables, there was marked variability in the ratings, with large standard deviations for most categories. Correlations between self-report measures are presented in Supporting Information Table 3. All correlations were weak to moderate.

### 3.1 Do people consistently interpret slow, gentle, caressing touch and hugs as communicating specific, positive emotions and social intentions?

Before investigating the role of context and person variables in the perception of emotions and intentions communicated by touch, we first tested the hypothesis that people would generally interpret slow, gentle, caressing touch and hugs as communicating positive (vs. negative) emotions and intentions. Valence (positive vs. negative) was the predictor of interest. Full model results and *N* are presented in Supporting Information Table 4; for these and all subsequent analyses, the best fitting models were the final models including the predictors of interest. As expected, we found that gentle touch and hugs were rated as eliciting more positive (*M* = 64.68, *SE* = 0.10) than negative (*M* = 7.59, *SE* = 0.10) emotions (*f*^2^ = 1.12) and conveying more positive (*M* = 54.32, *SE* = 0.10) than negative (*M* = 5.65, *SE* = 0.10) intentions (*f*^*2*^ = 1.15), with large effect sizes.^2^

### 3.2 Do people consistently interpret slow, gentle, caressing touch and hugs as more erotic (desire emotions and intentions), loving, and socially supportive if they have their partners in mind than if they have others in mind? Is there a difference between men and women in these effects?

We next investigated the impact of whom participants had had in mind (touch source) when they rated imagined touch (across both hugs and gentle touch) and how this varied by gender. We considered desire and love for emotions, and desire and support for intentions, and created a binary predictor variable for touch source (partner vs. other) from initially six categories (partner, friend, family member, stranger, no one in particular, someone else); groups were very similar in size with *N* = 6739 participants reporting having thought of their partner, and *N* = 5759 having thought of someone else in the emotions analysis, and *N* = 6766 participants having reported thinking of their partner, and *N* = 5826 of someone else in the intentions analysis (total *N* reduced due to missing data on the touch source question, and slightly different *N* between emotion and intention analyses given exclusions; see Section 2.4). We also entered gender (men, women) and the specific emotion/intention (love and desire for emotion; support and desire for intention) and all interaction terms as predictors of interest.

**Figure 3:**
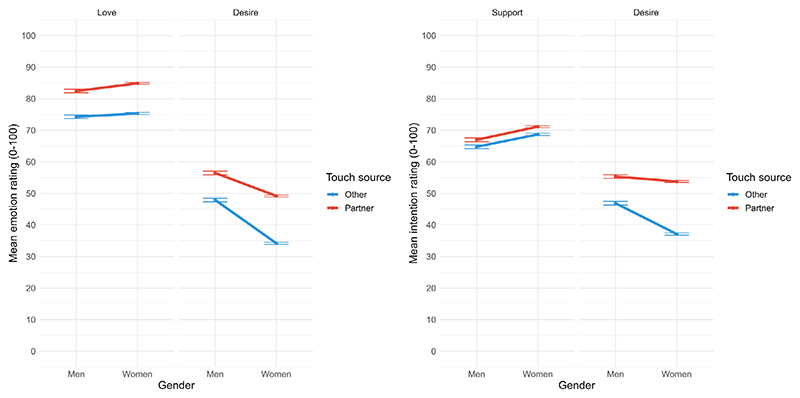
Emotion ratings for love and desire (left panel) and intention ratings for support and desire (right panel) by gender and touch source. Error bars denote ±1*SE* of the mean.

Full model results are presented in Table 2. Considering main effects, there was a significant effect of touch source for emotions (*f*^*2*^ = 0.07) and intentions (*f*^*2*^ = 0.04): Ratings were higher when participants had their partner (*M* = 67.63, *SE* = 0.23) versus someone else in mind (*M* = 56.33, *SE* = 0.25) for emotions, and their partner (*M* = 62.14, *SE* = 0.23) versus someone else in mind (*M* = 53.63, *SE* = 0.25) for intentions, supporting Hypothesis 2. Furthermore, participants rated love and support higher than desire, and women gave lower ratings than men overall (see Figure 3).

Because participants chose whom to hold in mind when rating imagined touch, person variables associated with interpersonal relating might have influenced participants’ selected touch source. Therefore, in an exploratory analysis (not pre-registered), we examined whether attachment style predicted touch source. Interestingly, attachment anxiety and avoidance scores predicted whether people thought of a partner or someone else, with higher scores associated with greater odds of thinking of someone else (see Supporting Information).

The three-way interaction between gender, touch source, and specific category was significant for both emotions (*f*^2^ < 0.001) and intentions (*f*^2^ < 0.001). Specific contrasts (Bonferroni-corrected) revealed that both women and men gave lower ratings for love and desire (emotions) if they had someone else versus their partner in mind (men: contrast = −8.36, *SE* = 0.67, *p* < .001; women: contrast = −12.27, *SE* = 0.39, *p* < .001) and rated support and desire (intentions) lower if they had someone else versus their partner in mind (men: contrast = −5.34, *SE* = 0.67, *p* < .001; women: contrast = −9.55, *SE* = 0.39, *p* < .001). This finding was contrary to our prediction (Hypothesis 2.1) regarding this effect occurring in women only. However, women did give lower ratings for desire generally than did men for emotions (contrast = −10.48, *SE* = 0.47, *p* < .001) and intentions (contrast = −5.64, *SE* = 0.49, *p* < .001).

Acknowledging the heterogeneous nature of the ‘other’ category, we also explored (analysis not pre-registered) possible differences between common sources of love and support by contrasting partner, family member, and friend for love (emotion) and support (intention) by gender. For love, the interaction between touch source and gender was significant (*χ*^*2*^(2) = 26.62, *p* < .001): Both women and men reported feeling most love when they had had their partner in mind, followed by family member and then friend (see Supporting Information Table 5 for full model results). All Bonferroni-corrected contrasts were significant for women, but friend and family member touch sources did not differ significantly for men. Regarding support, there were no significant effects of touch source, gender, or their interaction (*χ*^*2*^(2) = 4.70, *p* = .095); see Supporting Information Figure 1 for these exploratory results.

### 3.3 Do people consistently interpret slow, gentle, caressing touch as more erotic (desire emotions and intentions) than a hug, and particularly when they have their partners in mind?

**Figure 4:**
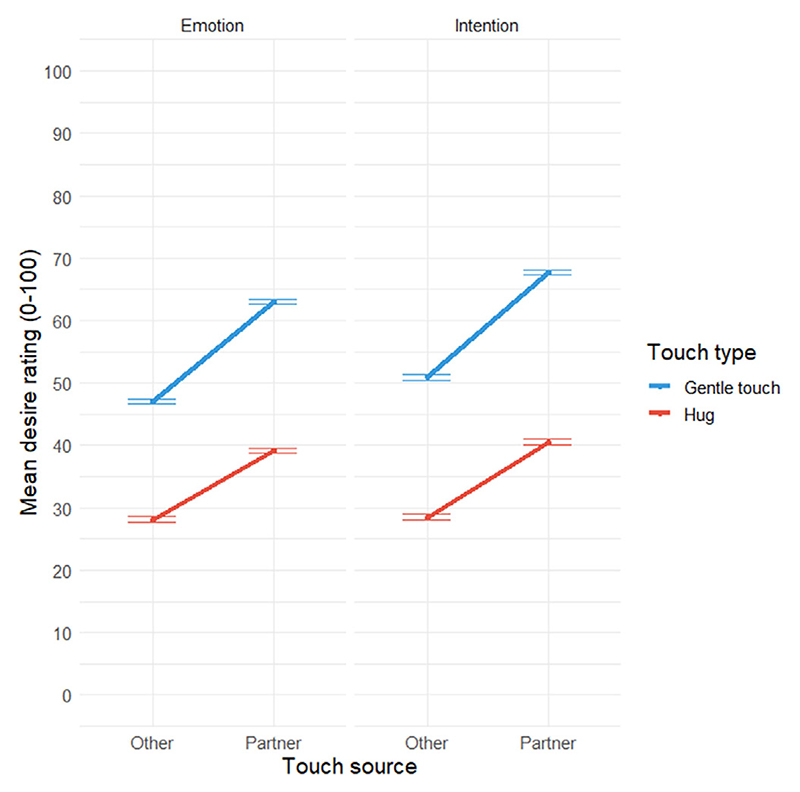
Emotion (left panel) and intention (right panel) ratings for desire by touch type and touch source. Error bars denote ±1*SE* of the mean.

To test Hypothesis 3, we examined the effects of type of touch, touch source, and their interaction on ratings for desire only (separately for emotion and intention). Full model results are presented in Supporting Information Table 6 (fit was best in full models). Confirming the first part of the question, gentle touch was rated as significantly more erotic than hugs for both emotions (*f*^2^ = 0.13; *M* = 55.70, *SE* = 0.27 for slow, gentle touch, and *M* = 34.04, *SE* = 0.27 for hugs) and intentions (*f*^2^ = 0.16; *M* = 59.92, *SE* = 0.28 for slow, gentle touch, and *M* = 34.99, *SE* = 0.28 for hugs). Furthermore, desire was rated more highly if participants had their partner versus someone else in mind. These main effects were further qualified by a significant interaction between touch type and touch source for both emotions (*f*^2^ = 0.001) and intentions (*f*^2^ = 0.001); see Figure 4. While all Bonferroni-corrected contrasts were significant, the difference between hug and gentle touch was greater if participants had had their partner versus someone else in mind for both emotions (partner contrast = −23.98, *SE* = 0.37, *p* < .001; other contrast = −18.96, *SE* = 0.40, *p* < .001) and intentions (partner contrast = −27.11, *SE* = 0.38, *p* < .001; other contrast = −22.40, *SE* = 0.40, *p* < .001), supporting Hypothesis 3: Gentle touch was rated as eliciting and conveying more desire when participants had thought of their partner as the source of the touch.

### 3.4 Is the communication of specific, positive emotions and social intentions via slow, gentle caressing touch and hugs predicted by individual differences in attachment, and positive recollection of childhood touch?

We ran analyses separately for attachment and childhood touch. While the two concepts were significantly related (see Supporting Information), correlations between positive childhood touch and attachment anxiety (*r* = −0.07, *p* < .001) and attachment avoidance (*r* = −0.22, *p* < .001) were weak, warranting us to examine positive childhood touch and attachment as separate constructs. We did not anticipate differences between types of touch, as both are used to signal closeness and support (Jakubiak & Feeney, 2016; Morrison, 2016), and so tested our hypotheses across both types of touch in the analyses, as pre-registered. However, as an exploratory analysis (not pre-registered), we also repeated analyses for hugs and gentle touch separately – patterns of effects were identical to those in the pre-registered analysis except that, for emotions only, all ratings were higher for gentle touch compared to hugs (see Supporting Information Figure 2).

#### 3.4.1 Attachment style

**Figure 5:**
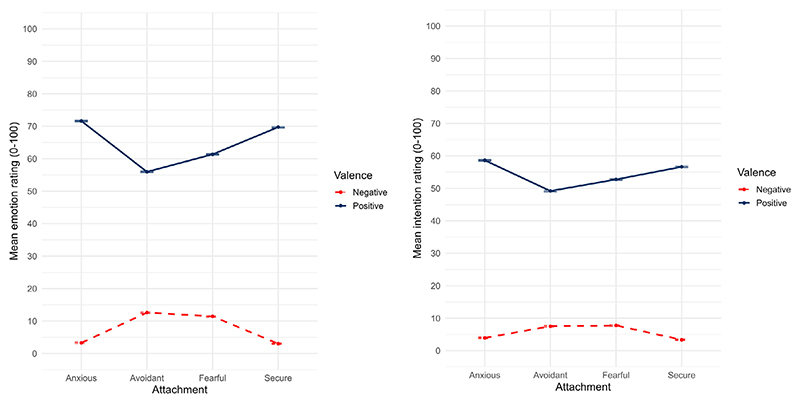
Mean touch ratings for positive and negative emotions (left panel) and intentions (right panel), by attachment styles. Note that attachment styles are marginal means at +1*SD* anxiety/−1*SD* avoidance for anxious, −1*SD* anxiety/+1*SD* avoidance for avoidant, +1*SD* anxiety/+1*SD* avoidance for fearful, and −1*SD* anxiety/−1*SD* avoidance for secure attachment, meaning participants were not assigned to attachment categories. Error bars show ±1*SE* of the mean.

Valence, attachment anxiety, attachment avoidance, and all interaction terms were examined as predictors of interest. All touch ratings were entered as the outcome. Full model results are presented in Table 3, and model fit was best for full models. There was a significant interaction between valence, attachment anxiety, and attachment avoidance for both emotions (*f*^2^ = 0.002) and intentions (*f*^2^ < 0.001). We probed this interaction by examining effects of valence at marginal means estimated at four combinations of dimensional attachment anxiety (−1*SD*, +1*SD*) and avoidance (−1*SD*, +1*SD*) scores (Aiken & West, 1991; Preacher et al., 2006), an approach which allows us to conceptualise attachment dimensions as ‘categories’ (Bartholomew & Horowitz, 1991) *without grouping* individuals. As outlined in Section 2, −1*SD* on both anxiety and avoidance dimensions was labelled as ‘secure attachment’, +1*SD* anxiety/−1*SD* avoidance as ‘anxious attachment’, −1*SD* anxiety/+1*SD* avoidance as ‘avoidant attachment’, and +1*SD* on both dimensions as ‘fearful attachment’. These marginal means are presented by valence in Figure 5. Bonferroni-corrected planned contrasts (with secure attachment as reference category) are presented in Supporting Information Table 7. Higher avoidant and fearful attachment scores were associated with higher ratings of negative emotions and intentions than secure attachment scores (*p*s < .001), while there was no difference between secure and anxious attachment scores. Furthermore, avoidant and fearful attachment scores were associated with lower ratings for positive emotions and intentions than secure attachment, while anxious attachment scores were associated with higher ratings for positive emotions and intentions than secure attachment scores (all *p*s < .001). Thus, part 1 of Hypothesis 4 was partially supported for avoidant and fearful, but not anxious sub-types of insecure attachment. Notably, findings for anxious and avoidant/fearful attachment went in opposite directions. We repeated the analysis with the same steps for only the people who had thought of their partner (exploratory question in pre-registration). Results fully mirrored those in the full sample (see Supporting Information Table 8).

To examine the discriminability question, we computed a ‘distinctiveness’ score. This particular outcome measure was not pre-registered but was needed to be able to address the ‘distinct’ part of the hypothesis (H4.2). Separately for emotions and intentions, we computed absolute difference scores for the different emotions/intention categories (e.g. love vs. happiness, love vs. desire, love vs. annoyance, love vs. anger, love vs. fear and repeated for all combinations) and took the average of all these absolute differences as the outcome. Higher scores therefore denoted greater differences, that is, distinctions between emotion categories and (separately) intention categories. As the outcome variables were negatively skewed, we ran a multiple regression analysis with bootstrapping (1000 replications). For both emotions and intentions, attachment anxiety and avoidance (but not their interaction) were significantly associated with distinctiveness – but in opposite directions (see Table 4 for full model results). While higher attachment avoidance was associated with lower distinctiveness (partial *η*^2^ = 0.015 [95% CIs = 0.012; 0.019] for emotions and 0.008 [95% CIs = 0.005; 0.010] for intentions), supporting this part of the hypothesis, attachment anxiety was associated with greater distinctiveness of categories (partial *η*^*2*^ = 0.001 [95% CIs = 0.000; 0.001] for emotions and 0.002 [95% CIs = 0.001; 0.004] for intentions). This finding is thus in contrast with this part of the hypothesis, but in line with results for part 1 (H4.1); that is, anxiety and avoidance showed opposite patterns. When examining these associations only in participants who had thought of their partner, results were very similar (see Supporting Information and Supporting Information Table 9): Here, the attachment by avoidance interaction was significant, showing that avoidant/fearful attachment scores were associated with lower distinctness of emotions and intentions, while anxious and secure attachment scores were associated with greater distinctness.

#### 3.4.2 Positive childhood touch

Valence, positive childhood touch, and their interaction were examined as predictors of interest. We considered all touch ratings and ran the analysis across type of touch. There was a significant interaction between valence and positive childhood touch for both emotions (*f*^2^ = 0.009) and intentions (*f*^2^ = 0.004; see Supporting Information Table 10 for full model results). Although all planned contrasts (comparing positive vs. negative valence at −1*SD*, mean, and +1*SD* of positive childhood touch scores) were significant, greater positive childhood touch was related to higher ratings for positive and lower ratings for negative emotions and intentions, in line with the first part of the hypothesis (H4.1; see Supporting Information Figure 3). Furthermore, in the distinctiveness analysis, greater positive childhood touch was significantly associated with greater discrimination between emotion categories (partial *η*^2^ = 0.004 [95% CIs = 0.003; 0.006]) and intention categories (partial *η*^2^ = 0.003 [95% CIs = 0.002; 0.005]), supporting part 2 of the hypothesis (H4.2; see Supporting Information Table 11). Taken together, more positive reported childhood touch was associated with more positive emotions and intentions, and greater distinctiveness between emotion and intention categories when rating imagined social touch.

## 4 Discussion

The present study sought to investigate how relational context and person variables shape the meaning associated with imagined social touch. We found that, overall, gentle, caressing touch and hugs were rated as evoking and conveying more positive than negative emotions and intentions, supporting our first hypothesis (H1). Considering the relational context, specifically whom participants had had in mind when rating the touch, we found that ratings of love and desire (emotions) and love and support (intentions) were higher when participants had had their partner (vs. someone else) in mind, supporting H2, and that these findings were moderated by gender: While both men and women rated love, support, and desire more strongly when they had had their partner (vs. someone else) in mind, women gave lower ratings for desire than did men for emotions and intentions in general (partially supporting H2.1). Focusing specifically on erotic arousal, we further found that desire ratings were higher for gentle touch compared to hugs, particularly when participants had had their partner (vs. someone else) in mind (supporting H3). Lastly, considering individual differences, we found partial support for H4, in that results diverged for anxious and avoidant attachment: Avoidant attachment scores were associated with lower ratings for positive emotions and intentions, and less distinctiveness between categories, compared to secure attachment scores, whereas anxious attachment scores were associated with higher ratings for positive emotions and intentions and greater discriminability between categories compared to secure attachment scores. Positive childhood touch was associated with more positive emotions and intentions, and with greater distinctness between emotion and intention categories when rating imagined social touch.

Considering hugs and slow, gentle touch together, we first hypothesised that these forms of touch would generally be rated as conveying positive rather than negative emotions and intentions. Gentle stroking has previously been found to be decoded as the emotions love (Hertenstein et al., 2006; McIntyre et al., 2022; Nguyen et al., 1975) and desire (Kirsch et al., 2018; Nguyen et al., 1975), and the intention to communicate support (Kirsch et al., 2018). Hugging has been associated with positive mood (Packheiser et al., 2024), patting with playfulness (Nguyen et al., 1975), and holding or light squeezing with gratitude (McIntyre et al., 2022). We used the emotion and intention categories in Kirsch et al. (2018) and presented the same synonyms for each to help clarify concepts. Though we explored specific emotions and intentions regarding different relational contexts, we were initially interested in general valence effects of gentle touch and hugs, rather than examining which specific emotions and intentions were associated with touch. We found that, across the two types of touch, participants gave significantly higher ratings for feeling positive (vs. negative) emotions when imagining the touch, and for positive (vs. negative) intentions being conveyed by this touch. Our findings support the idea that, in general, hugs and gentle touch are associated with positive meaning.

This interpretation of touch as positive has been conceptualised as a critical pathway to well-being in close relationships (Jakubiak & Feeney, 2017). Jakubiak and Feeney (2017) propose that seeing touch as affectionate and prosocial leads to cognitive-relational changes, including enhanced felt security, which facilitates closeness and increases cognitions that support is available when needed. In other words, this model highlights the link between the meaning associated with touch, secure attachment, and greater well-being. Jakubiak and Feeney’s model does not contrast close relationships with other relationships. When we contrasted partner, friend, and family member, touch imagined from all these close relationship contexts was similarly associated with support (and there were no gender differences).

As participants were asked to indicate whom they had had in mind *after* they completed the touch ratings, it is possible that people naturally chose to think of supportive and loving others. There was some evidence that attachment styles were associated with whom people had in mind, with higher attachment anxiety and avoidance linked to a greater likelihood of holding someone other than a partner in mind. However, we did not ask about people’s relationship histories or current relationship status, and so it is possible that more insecurely attached participants did not have a partner to think about. To counteract these issues, we could have varied imagined touch source more systematically by asking certain participants to imagine their partner (if they had one, or by recruiting only people currently in a relationship) and others to imagine somebody else, but this was beyond the possibilities of this large-scale survey. Indeed, it would not have been in keeping with the Touch Test’s aims to explore naturally occurring attitudes towards touch and touch experiences in the general population.

We did find several effects that were strongest in partner contexts. Love and desire (the latter more so for men than women) were rated more highly when participants had their partner (vs. someone else) in mind, and this was still the case for love when we contrasted partner with friend and family member. Regarding desire, we examined hugs and gentle stroking touch separately because adult partners stroke each other at speeds which are optimal for activating CT fibres (Croy et al., 2016) and this CT-optimal, gentle touch (compared to non-CT-optimal touch) is perceived as erotically arousing (Bendas et al., 2017) in close relationship contexts (Panagiotopoulou et al., 2018). Building on the role of CT-mediated touch in shaping arousal, we found, in line with our hypothesis, that gentle touch was rated as significantly more erotic than hugs for both emotions and intentions. Furthermore, gentle touch was most arousing, and decoded as conveying desire, when participants had had their partner (vs. someone else) in mind. A limitation here is that previous research explored touch to erogenous versus non-erogenous zones (Heslin et al., 1983) and, while dissociable from stroking speed in Panagiotopoulou et al.’s (2018) study, it would have been useful to ask which body parts participants were imagining being touched. Not asking participants to indicate imagined touch location is a more general limitation of the present research. Touch meaning may vary depending on where touch is applied, and this may interact with relationship closeness (Heslin et al., 1983; McIntyre et al., 2022; Suvilehto et al., 2015), and person factors such as attachment styles, especially if the location imagined is more or less intimate. Furthermore, arousal ratings for touch to one’s own body have been found to correspond with arousal for touch when imagining one’s partner’s body (Maister et al., 2020), and examining meaning of touch in terms of evoking and conveying arousal would be interesting to explore in reciprocal interpersonal tasks. Nevertheless, our findings demonstrate that closeness and intimacy with an imagined toucher shape the meaning of slow, gentle touch and hugs, with differences between men and women in these effects.

Most previous research has investigated the role of person variables in shaping the affective quality of touch (e.g., perceived pleasantness; see Sailer & Ackerley, 2019; Suvilehto et al., 2015) or its effects in terms of reducing negative affective states (e.g. Krahé et al., 2016; von Mohr et al., 2018) and increasing well-being (Debrot et al., 2020). We examined both positive childhood touch and differences in adult attachment styles as potential moderators of touch meaning. More positive childhood touch was linked to more positive emotions and intentions associated with touch, and more discrimination between emotion and intention categories. Of note, positive childhood touch was reported retrospectively, and the scale, though it had good internal consistency, comprised only two items taken from a longer, validated scale. Thus, findings should be interpreted with caution.

Examining attachment styles (the development of which is undoubtedly influenced by positive childhood touch, although the correlation between the two constructs was weak in the present study), we hypothesised that insecure attachment would be associated with less positive and distinct emotions and intentions. Imagined rather than experienced touch may be particularly well-suited for illuminating differences in touch meaning by attachment styles. It draws on people’s episodic memories and imagination in the absence of actual sensory input (Cao et al., 2018), and is perhaps even more susceptible to individual differences than experienced touch, usually administered with specific instructions and within constrained contexts, would be (though see daily diary studies for more ecologically valid ways of measuring experienced touch, e.g. Carmichael et al., 2021). Higher attachment avoidance is linked with higher levels of mistrust in others, less desire for, and enjoyment of, touch (Carmichael et al., 2021), less craving for touch during the Covid-19 pandemic (von Mohr et al., 2021), maintaining interpersonal distance, including touching one’s partner less often in daily life (Carmichael et al., 2021), and – as research also using Touch Test data showed – a greater tendency to avoid tactile treatments in health settings (Vafeiadou et al., 2022). In this paper, our prediction regarding attachment avoidance was confirmed, with attachment avoidance linked to lower ratings for positive emotions and intentions, and less rated distinctiveness between categories. Interestingly, evidence is emerging that in unforced settings (i.e. outside laboratory settings where people are required to experience touch), more avoidantly attached people do report relationship benefits upon receiving touch from their partner in much the same way as less avoidantly attached people (Carmichael et al., 2021). Future research could seek to further untangle touch meaning from touch effects in relation to attachment avoidance.

Higher attachment anxiety is generally related to a more ambivalent stance, namely simultaneously desiring closeness and fearing rejection and abandonment (Mikulincer et al., 2003). Higher attachment anxiety has also been associated with reduced discrimination between ‘affective’ CT-optimal stroking touch and more ‘neutral’ non-CT-optimal touch when rating pleasantness (Krahé et al., 2018). In our study, we found that attachment anxiety was linked to more positive emotions and intentions and greater discrimination between categories. Regarding the former, recent research also found that higher attachment anxiety was related to greater craving for touch during the Covid-19 pandemic (von Mohr et al., 2021), greater self-reported desire for, and greater enjoyment of, (imagined) touch, and more benefits of actually-received touch from the romantic partner in terms of perceived responsiveness and closeness (Carmichael et al., 2021). Contrary to the more general ambivalence towards others associated with attachment anxiety, social touch may be a powerful positive signal of closeness and connection and carry a positive meaning for more anxiously attached people, which promotes relationship quality (Carmichael et al., 2021). Though they did not measure individual differences in attachment style, a recent study found links of touch during sleep and subsequent morning mood with positive relationship quality (spousal enjoyment) at the end of the day (Roberts et al., 2022). With these benefits in mind, and our findings of positive touch meaning in relation to higher attachment anxiety, a practical implication is to encourage the use of social touch, especially in close relationship contexts.

Regarding greater discrimination between emotion and intention categories associated with attachment anxiety, it is possible that hypervigilance and ‘hyperactivating strategies’ (Mikulincer et al., 2003) linked with attachment anxiety make social signals of reassurance especially salient and facilitate a more nuanced interpretation of them, even when imagined. While this latter finding is at odds with research examining the affective quality of touch, it can be explained in relation to Sailer and Leknes’s (2022) model, in which touch meaning is compared with goals to impact affective experience. Positive touch meaning and the relational goal to seek closeness and reassurance may reduce differences in perceived pleasantness between what are ultimately two forms of social touch.

In contrast to previous research in which participants indicated which emotions and intentions they thought were conveyed by the toucher (Kirsch et al., 2018), the Touch Test asked participants to indicate which emotions *they themselves felt* when imagining social touch. While we have conceptualised both emotions (evoked) and intention (communicated) ratings as interpreting and ascribing meaning to touch, it could be argued that emotion ratings reflect the affective quality of touch rather than meaning per se. However, as we have discussed, these concepts inform each other in likely reciprocal ways (Sailer & Leknes, 2022) and are difficult to untangle from survey data. Given that emotion and intention results mirrored each other for most analyses, and intention categories included concepts such as praise and support, which are not emotion terms, it is unlikely that participants approached emotions and intentions in entirely different ways. However, the similarities between the two do indicate that participants might not have distinguished between emotions and intentions in the way it was intended.

Too few participants from outside the United Kingdom completed the survey to address differences across countries or world regions, and so only participants resident in the United Kingdom were included in the current study. Fascinating research is emerging on regional and cultural factors, such as air temperature and collectivism, in shaping touch behaviour (Sorokowska et al., 2021) and it would be invaluable to study such factors in relation to touch meaning. Furthermore, this study was correlational in nature, and so we cannot make any claims regarding causality. In addition, all measures were self-report measures and some measures were unvalidated, as they were shorter versions of existing scales. These are limitations of the present research and reflect the compromises inherent in designing large-scale surveys. Lastly, we studied imagined touch rather than directly-cexperienced touch. As well as being a useful proxy for experienced touch (being able to elicit feelings of pleasantness, e.g. Panagiotopoulou et al., 2018), imagined touch also has advantages over and above experienced touch in terms of reaching people who may not have access to direct touch, or enhancing digital communication (Saramandi et al., 2023). An additional advantage relates to the perceived permissibility and acceptability of touch, which is influenced by touch source (Suvilehto et al., 2015) and culture (Burleson et al., 2019). Imagined touch may be more acceptable to people, as they can maintain full control over what they think about, compared to experienced touch in certain contexts. However, a limitation of imagined touch is that we do not know exactly how people approached the task, and how they imagined the touch (e.g. whether they saw concrete mental images or imagined touch in more abstract ways, and the amount of detail imagined; Cao et al., 2018). Furthermore, it is possible that people responded in ways that reflect social norms around recognising hugs and gentle touch as positive rather than how they themselves would feel or interpret such touch.

Using an imagined touch paradigm and recruiting a big sample from the general population, with a large age range (especially compared to laboratory studies often conducted in student samples), allowed us to explore contextual and person variables emerging from research on experienced touch with more power and statistical rigour than is sometimes possible in laboratory settings. We were able include relevant covariates and examine complex interaction effects, including gender differences. We confirmed certain findings (the overwhelmingly positive meaning ascribed to gentle touch and hugs), and discovered novel insights regarding attachment styles, showing that attachment anxiety and avoidance are differentially associated with discriminability of specific emotions and intentions elicited and conveyed by imagined touch. These findings can now inform studies systematically exploring the influence of contextual and person variables on directly experienced touch to enhance our understanding of the meaning of touch in social interactions, and the promotion of well-being through touch.

## Supplementary Material

Supplementary material

## Figures and Tables

**Table 1:** Demographic characteristics.

		*N*	Mean	*SD*	Min	Max
Age		23,428	57.05	13.92	19	94
			%			
Gender	Men	5,909	25.20			
	Women	17,536	74.80			
Ethnicity	White	22,426	95.70			
	Black	119	0.50			
	Asian	273	1.20			
	Mixed/multiple	349	1.50			
	Other background/prefer not to say	263	1.10			
Sexuality	Heterosexual	20,989	89.50			
	Bisexual	976	4.17			
	Gay or lesbian	745	3.18			
	Prefer not to say	306	1.30			
	Prefer to self-describe	435	1.86			
Recent touch experience/last time touched	Within last hour	5,927	25.30			
	Last day or less	10,765	45.90			
	Last week or less	4,455	19.00			
	In the last month	1,378	5.90			
	Over a month ago	666	2.80			
	Over a year ago	284	1.20			
Completed before first UK lockdown began on 23 March 2020	Yes	23,175	98.92			
	No	253	1.08			

*Note*. Due to missing responses, not all categories add up to N = 23,475.

**Table 2:** Effects of person in mind, gender, and specific category on emotions and intentions.

		Emotions (*N* = 12,498)						Intentions (*N* = 12,593)					
		*b*	*SE*	*p*	95% CI			*b*	*SE*	*p*	95% CI		
Intercept		21.78	1.88	<.001	18.10	25.45		29.28	1.90	<.001	25.56	32.99	
Covariates													
	>1 Month ago	1.58	1.74	.364	−1.83	4.98		1.68	1.75	.337	−1.75	5.11	
Last time touched (Over a year ago = reference category)													
	≤Last month	1.60	1.63	.326	−1.59	4.79		2.11	1.64	.198	−1.10	5.32	
	≤Last week	4.18	1.53	.006	1.17	7.18		2.96	1.54	.056	−0.07	5.98	
	≤Last day	4.52	1.51	.003	1.57	7.48		4.16	1.52	.006	1.18	7.14	
	Last hour	4.66	1.53	.002	1.65	7.66		4.98	1.54	.001	1.95	8.00	
Ability to empathise		0.34	0.04	<.001	0.25	0.42		0.40	0.04	<.001	0.31	0.48	
Attitudes to intimate touch		10.83	0.19	<.001	10.47	11.19		5.94	0.19	<.001	5.57	6.30	
Week since start of 2020		0.13	0.06	.034	0.01	0.25		0.10	0.06	.098	−0.02	0.22	
Interoceptive sensibility		1.18	0.20	<.001	0.80	1.56		0.68	0.20	.001	0.30	1.07	
Predictors of interest													
Touch source		−8.16	0.79	**<.001**	−9.71	−6.62		−2.22	0.83	**.007**	−3.84	−0.60	
Gender		2.42	0.64	**<.001**	1.16	3.68		4.17	0.67	**<.001**	2.84	5.49	
Specific category		−25.98	0.60	**<.001**	−27.15	−24.81		−11.65	0.69	**<.001**	−13.01	−10.29	
Touch source × Gender		−1.38	0.90	.126	−3.16	0.39		−0.24	0.95	.801	−2.10	1.62	
Touch source × Specific category		−0.40	0.84	.636	−2.05	1.25		−6.24	0.98	**<.001**	−8.16	−4.32	
Gender × Specific category		−9.69	0.68	**<.001**	−11.02	−8.36		−5.72	0.79	**<.001**	−7.27	−4.17	
Touch source × Gender × Specific category		−5.04	0.97	**<.001**	−6.95	−3.13		−7.94	1.13	**<.001**	−10.15	−5.73	
Participant (random intercept)		197.84	4.35		189.49	206.56		151.84	4.46		143.34	160.85	
Intercept residual		548.44	4.01		540.65	556.35		742.69	5.40		732.17	753.36	

*Note*: Significant findings of interest are highlighted in bold. For emotions, full model ICC = 0.265, *SE* = 0.004, 95% CI = 0.256 −0.275, log-likelihood = −234,080.52, AIC = 468,199, BIC = 468,366.6; model with covariates: ICC = 0.124, *SE* = 0.004, 95% CI = [0.117, −0.131], log-likelihood = −357,746.38, AIC = 715,516.8, BIC = 715,627.1; intercept-only model: ICC = 0.233, *SE* = 0.004, 95% CI = [0.226, −0.240], log-likelihood = −441,201.39, AIC = 882,408.8, BIC = 882,437. For intentions, full model ICC = 0.170, *SE* = 0.005, 95% CI = [0.161, −0.179], log-likelihood = −241,642.9, AIC = 483,323.8, BIC = 483,491.5; model with covariates: ICC = 0.128, *SE* = 0.004, 95% CI = [0.121, −0.135], log-likelihood = −358,064.68, AIC = 716,153.4, BIC = 716,263.8; intercept-only model: ICC = 0.175, *SE* = 0.003, 95% CI = [0.168, −0.182], log-likelihood = −439,795.89, AIC = 879,597.8, BIC = 879,626. Abbreviations: ICC, Intraclass correlation coefficient; *SE*, standard error; CI, confidence interval; AIC, Aikake information criterion; BIC, Bayesian information criterion.

**Table 3:** Interaction between valence, attachment anxiety, and attachment avoidance on ratings for emotions and intentions.

		Emotions (*N* = 18,161)						Intentions (*N* = 18,281)					
		*b*	*SE*	*p*	95% CI	* *		*b*	*SE*	*p*	95% CI		
Intercept		−11.84	0.90	<.001	−13.61	−10.07		−7.76	1.02	<.001	−9.75	−5.77	
Covariates													
	>1 Month ago	−0.59	0.87	.499	−2.29	1.11		−0.52	0.98	.594	−2.43	1.39	
Last time touched (Over a year ago = reference category)													
	≤Last month	−1.13	0.81	.161	−2.72	0.45		−1.24	0.91	.172	−3.03	0.54	
	≤Last week	−0.64	0.76	.397	−2.13	0.85		−0.83	0.86	.335	−2.51	0.85	
	≤Last day	0.08	0.75	.912	−1.39	1.55		−0.12	0.85	.884	−1.78	1.54	
	Last hour	0.60	0.76	.434	−0.90	2.09		0.32	0.86	.712	−1.37	2.00	
Ability to empathise		0.20	0.02	<.001	0.15	0.24		0.21	0.02	<.001	0.16	0.26	
Attitudes to intimate touch		3.74	0.09	<.001	3.56	3.92		2.21	0.10	<.001	2.01	2.42	
Week since start of 2020		0.06	0.03	.053	0.00	0.12		0.06	0.03	.074	−0.01	0.13	
Interoceptive sensibility		0.17	0.10	.068	−0.01	0.36		0.30	0.11	.004	0.10	0.51	
Predictor of interest													
Valence		57.04	0.11	**<.001**	56.83	57.25		48.64	0.10	**<.001**	48.44	48.84	
Attachment anxiety		−0.16	0.07	**.022**	−0.30	−0.02		0.15	0.07	**.042**	0.01	0.29	
Attachment avoidance		3.60	0.09	**<.001**	3.43	3.76		1.62	0.09	**<.001**	1.44	1.80	
Valence × Attachment anxiety		1.44	0.08	**<.001**	1.29	1.59		0.83	0.07	**<.001**	0.69	0.97	
Valence × Attachment avoidance		−8.46	0.09	**<.001**	−8.64	−8.29		−4.33	0.08	**<.001**	−4.49	−4.16	
Attachment anxiety × Attachment avoidance		−0.20	0.05	**<.001**	−0.30	−0.10		−0.05	0.05	.368	−0.15	0.06	
Valence × Attachment anxiety × Attachment avoidance		0.70	0.06	**<.001**	0.59	0.81		0.27	0.05	**<.001**	0.16	0.37	
Participant (random intercept)		61.86	1.21		59.53	64.28		97.23	1.51		94.32	100.23	
Intercept residual		630.46	2.00		626.56	634.38		554.80	1.75		551.38	558.24	

*Note*: Significant findings of interest are highlighted in bold. For emotions, full model ICC = 0.089, *SE* = 0.002, 95% CI = [0.086, 0.093], log-likelihood = −1,017,836.9, AIC = 2,035,712, BIC = 2,035,907; model with covariates: ICC = 1.75 × 10^−16^, *SE* = 0, 95% CI = [1.75 × 10^−16^, 1.75 × 10^−16^], log-likelihood = −1,111,763.2, AIC = 2,223,550, BIC = 2,223,674; intercept-only model: ICC = 0.005, *SE* = 0.001, 95% CI = [0.003, 0.007], log-likelihood = −1,360,813.1, AIC = 2,721,632, BIC = 2,721,664. For intentions, full model ICC = 0.149, *SE* = 0.002, 95% CI = [0.145, 0.153], log-likelihood = −1,014,025.5, AIC = 2,028,089, BIC = 2,028,285; model with covariates: ICC = 0.035, SE = 0.001, 95% CI = [0.032, 0.037], log-likelihood = −1,096,085, AIC = 2,192,194, BIC = 2,192,318; intercept-only model: ICC = 0.043, *SE* = 0.001, 95% CI = [0.041, 0.046], log-likelihood = −1,341,257.1, AIC = 2,682,520, BIC = 2,682,552. Abbreviations: ICC, intraclass correlation coefficient; *SE*, standard error; CI, confidence interval; AIC, Aikake information criterion; BIC, Bayesian information criterion.

**Table 4:** Bootstrapped regression analysis for effects of attachment anxiety and attachment avoidance on distinctness of emotions and intentions.

			Emotions (*N* = 18,161)						Intentions (*N* = 18,281)					
			*b*	*SE*	*p*	95% CI			*b*	*SE*	*p*	95% CI		
Intercept			16.56	1.19	<.001	14.24	18.88		16.65	1.13	<.001	14.44	18.86	
Covariates														
	Last time touched (Over a year ago = reference category)	>1 Month ago	−0.81	1.11	.469	−2.99	1.38		0.81	1.10	.460	−1.34	2.96	
		≤ Last month	−1.06	1.05	.310	−3.12	0.99		1.26	1.05	.228	−0.79	3.32	
		≤Last week	0.24	1.03	.817	−1.78	2.26		2.01	0.99	.043	0.07	3.95	
		≤ Last day	0.78	1.02	.444	−1.21	2.77		2.79	0.99	.005	0.86	4.73	
		Last hour	1.49	1.02	.143	−0.51	3.49		3.27	1.00	.001	1.32	5.22	
	Ability to empathise		0.26	0.02	<.001	0.22	0.30		0.32	0.02	<.001	0.28	0.36	
	Attitudes to intimate touch		4.30	0.10	<.001	4.10	4.50		2.59	0.10	<.001	2.40	2.78	
	Week since start of 2020		0.31	0.03	<.001	0.26	0.36		0.17	0.03	<.001	0.12	0.23	
	Interoceptive sensibility		0.75	0.10	<.001	0.55	0.95		0.62	0.10	<.001	0.43	0.81	
Predictors of interest	Attachment anxiety		0.17	0.06	**.003**	0.06	0.28		0.37	0.06	**<.001**	0.26	0.48	
	Attachment avoidance		−1.14	0.08	**<.001**	−1.29	−1.00		−0.86	0.08	**<.001**	−1.01	−0.71	
	Attachment anxiety × Attachment avoidance		−0.01	0.05	.806	−0.11	0.08		0.07	0.04	.102	−0.01	0.16	

*Note*. Significant findings of interest are highlighted in bold. CI, confidence interval.

## Data Availability

The analysis plan for this study was pre-registered on the OSF (https://osf.io/gvjqz) The dataset used in the analyses can be accessed here: https://osf.io/qt53j/

## Footnotes

1 We are aware that Cronbach’s alpha may underestimate reliability for two-item scales, and that other methods such as Spearman–Brown may be preferable. As we did not include the full original subscales and only have data for these two items, we report Cronbach’s alpha and acknowledge that the true internal consistency may have been higher.

2 Conditional *f*^2^ values were calculated in R using lme4 and sjPlot packages. For models with only one predictor of interest, the full model was compared to the covariates-only model. To isolate effects of individual predictors in models with several predictors of interest, the full model was compared to a reduced model without that specific predictor. Where specific predictors were also included in interaction terms, interaction terms including the predictor of interest were also removed from the reduced model given that interaction effects may influence effects of individual predictors. Where the effect of interest was a three-way interaction, only the three-way interaction term was removed from the reduced model; resulting effect sizes were very small.
