# Supplementary material for: The meaning of touch: Relational and individual variables shape emotions and intentions associated with imagined social touch
Source: Eur J Soc Psychol. Author manuscript; Available in PMC 2024 Dec 4. (PMC7616566; doi:10.1002/ejsp.3076)
Supplement: Supplementary material [file EMS197880-supplement-Supplementary_material.docx]

**Supporting information**

**Supplementary Table 1.** *Example data excerpt (independent variables and one example demographic variable only) for one participant to indicate the data layout.*

| **subj_id** | **touch_type** | **signal_type** | **valence** | **spec_outcome** | **age** |
| --- | --- | --- | --- | --- | --- |
| xxx | gentle_touch | emo | neg | 1 | 45 |
| xxx | gentle_touch | emo | neg | 2 | 45 |
| xxx | gentle_touch | emo | neg | 3 | 45 |
| xxx | gentle_touch | emo | pos | 1 | 45 |
| xxx | gentle_touch | emo | pos | 2 | 45 |
| xxx | gentle_touch | emo | pos | 3 | 45 |
| xxx | gentle_touch | msg | neg | 1 | 45 |
| xxx | gentle_touch | msg | neg | 2 | 45 |
| xxx | gentle_touch | msg | neg | 3 | 45 |
| xxx | gentle_touch | msg | pos | 1 | 45 |
| xxx | gentle_touch | msg | pos | 2 | 45 |
| xxx | gentle_touch | msg | pos | 3 | 45 |
| xxx | hug | emo | neg | 1 | 45 |
| xxx | hug | emo | neg | 2 | 45 |
| xxx | hug | emo | neg | 3 | 45 |
| xxx | hug | emo | pos | 1 | 45 |
| xxx | hug | emo | pos | 2 | 45 |
| xxx | hug | emo | pos | 3 | 45 |
| xxx | hug | msg | neg | 1 | 45 |
| xxx | hug | msg | neg | 2 | 45 |
| xxx | hug | msg | neg | 3 | 45 |
| xxx | hug | msg | pos | 1 | 45 |
| xxx | hug | msg | pos | 2 | 45 |
| xxx | hug | msg | pos | 3 | 45 |

*Note.* Subj_id = participant ID (redacted here); touch_type = gentle touch and hugs; signal_type = emotion (emo) and intention (msg); valence = negative (neg) and positive (pos); spec_outcome = specific emotion and intention, emo neg 1 = disgust, emo neg 2 = anger, emo neg 3 = fear, emo pos 1 = affection, emo pos 2 = joy, emo pos 3 = arousal, msg neg 1 = warning, msg neg 2 = aggression, msg neg 3 = fear, msg pos 1 = support, msg pos 2 = praise, msg pos 3 = arousal.

*Assumptions, transformation, and outliers:*

Assumptions of normality were checked using gg-plots, histograms, and tests of normality (e.g., Kolmogorov-Smirnov tests). For moderate skew or kurtosis, log-transformations or similar were applied. For extreme deviations from normality, we planned to use equivalent non-parametric tests (not necessary after inspecting the data). To deal with outliers in our analyses, we planned to use robust methods for multilevel analysis. However, as no small demographic subgroups were included, we did not exclude any outliers from analyses, and used maximum likelihood estimation instead of robust methods.

Individuals identified as having extreme values in group comparisons (i.e., 2.5*SD* from the group mean in normal or normalised distributions, or else the equivalent Interquartile Range) within a relatively small demographic subgroup (and therefore likely to have an excessive influence on the results of this group) were planned to be removed as outliers (but this did not need to be done).

**Supplementary Table 2.** *Descriptive statistics (mean, standard deviation, N) for all touch ratings.*

|  |  |  | **Mean** | ***SD*** | ***N*** |
| --- | --- | --- | --- | --- | --- |
| Hug | Emotion | Affection/love/intimacy | 77.89 | 25.13 | 23283 |
|  |  | Disgust/annoyance/irritation | 6.45 | 15.94 | 22853 |
|  |  | Joy/happiness/delight | 71.35 | 28.22 | 23235 |
|  |  | Anger/rage/fury | 3.46 | 11.95 | 22796 |
|  |  | Arousal/lust/desire | 33.44 | 32.85 | 22984 |
|  |  | Fear/terror/anxiety | 6.52 | 17.60 | 22812 |
|  | Intention | Aggression/intimidation/hostility | 3.48 | 12.87 | 22758 |
|  |  | Praise/compliment/reward | 54.90 | 30.40 | 23019 |
|  |  | Support/reassurance/encouragement | 73.02 | 24.13 | 23125 |
|  |  | Warning/caution/alarm | 5.05 | 14.10 | 22735 |
|  |  | Arousal/lust/desire | 34.45 | 32.46 | 22886 |
|  |  | Fear/terror/anxiety | 6.49 | 16.44 | 22727 |
| Gentle touch | Emotion | Affection/love/intimacy | 79.24 | 26.68 | 23330 |
|  |  | Disgust/annoyance/irritation | 13.10 | 23.12 | 22721 |
|  |  | Joy/happiness/delight | 69.50 | 30.54 | 23174 |
|  |  | Anger/rage/fury | 6.19 | 16.59 | 22469 |
|  |  | Arousal/lust/desire | 55.79 | 34.22 | 22974 |
|  |  | Fear/terror/anxiety | 10.11 | 21.98 | 22507 |
|  | Intention | Aggression/intimidation/hostility | 4.48 | 14.73 | 22718 |
|  |  | Praise/compliment/reward | 39.67 | 31.92 | 22959 |
|  |  | Support/reassurance/encouragement | 62.52 | 29.10 | 23156 |
|  |  | Warning/caution/alarm | 9.35 | 19.19 | 22672 |
|  |  | Arousal/lust/desire | 60.36 | 33.17 | 23017 |
|  |  | Fear/terror/anxiety | 6.02 | 15.88 | 22623 |

**Supplementary Table 3**. *Correlations between age, week completed, and questionnaire measures.*

|  | Age | IAS | Attitude to intimate touch | Positive childhood touch | ECR-anx | ECR_av | EQ | Week |
| --- | --- | --- | --- | --- | --- | --- | --- | --- |
| Age | 1 |  |  |  |  |  |  |  |
| IAS | 0.09* | 1 |  |  |  |  |  |  |
| Attitude to intimate touch | -0.07* | 0.21* | 1 |  |  |  |  |  |
| Pos childhood touch | -0.15* | 0.11* | 0.16* | 1 |  |  |  |  |
| ECR-anx | -0.15* | -0.11* | 0.09* | -0.07* | 1 |  |  |  |
| ECR_av | 0.06* | -0.22* | -0.38* | -0.22* | 0.10* | 1 |  |  |
| EQ-10 | -0.08* | 0.30* | 0.17* | 0.14* | 0.05* | -0.23* | 1 |  |
| Week | 0.02* | -0.00 | 0.02 | -0.00 | -0.01 | 0.01 | -0.01 | 1 |

*Note*. * = *p < .001*. IAS = Interoceptive Accuracy Scale; EC*R =* Experiences in Close Relationships; anx = anxiety; av = avoidance; EQ-10 = Empathy Quotient-10; Week – week since the start of the study (to account for Covid-19 pandemic).

**Supplementary Table 4**. *Effects of valence on touch ratings.*

|  |  | **Emotions (*N* = 18,224)** | | | | | **Intentions (*N* = 18,343)** | | | | |
| --- | --- | --- | --- | --- | --- | --- | --- | --- | --- | --- | --- |
|  |  | *b* | *SE* | *p* | [95% Conf. | Interval] | *b* | *SE* | *p* | [95% Conf. | Interval] |
| *Intercept* |  | -13.69 | 0.87 | < .001 | -15.40 | -11.98 | -9.35 | 0.98 | < .001 | -11.27 | -7.43 |
| *Covariates* |  |  |  |  |  |  |  |  |  |  |  |
| Last time touched  (Over a year ago = ref category) | >1 month ago | -0.61 | 0.87 | .483 | -2.31 | 1.09 | -0.52 | 0.98 | .593 | -2.43 | 1.39 |
|  | Last month or less | -1.21 | 0.81 | .136 | -2.80 | 0.38 | -1.32 | 0.91 | .148 | -3.10 | 0.47 |
|  | Last week or less | -0.69 | 0.76 | .366 | -2.18 | 0.80 | -0.87 | 0.86 | .312 | -2.55 | 0.81 |
|  | Last day or less | 0.05 | 0.75 | .942 | -1.42 | 1.53 | -0.17 | 0.84 | .836 | -1.83 | 1.48 |
|  | Last hour | 0.64 | 0.76 | .398 | -0.85 | 2.13 | 0.30 | 0.86 | .728 | -1.38 | 1.97 |
| Ability to empathise |  | 0.24 | 0.02 | < .001 | 0.20 | 0.28 | 0.25 | 0.02 | < .001 | 0.21 | 0.30 |
| Attitudes to intimate touch |  | 4.12 | 0.09 | < .001 | 3.95 | 4.29 | 2.56 | 0.10 | < .001 | 2.37 | 2.75 |
| Week since start of 2020 |  | 0.05 | 0.03 | .123 | -0.01 | 0.11 | 0.05 | 0.03 | .148 | -0.02 | 0.12 |
| Interoceptive sensibility |  | 0.13 | 0.09 | .185 | -0.06 | 0.31 | 0.24 | 0.11 | .023 | 0.03 | 0.45 |
| *Predictors of interest* |  |  |  |  |  |  |  |  |  |  |  |
| Valence |  | 57.09 | 0.11 | **< .001** | 56.87 | 57.30 | 48.67 | 0.10 | **< .001** | 48.47 | 48.87 |
| *Participant* (random intercept) |  | 60.28 | 1.22 |  | 57.93 | 62.73 | 97.32 | 1.51 |  | 94.39 | 100.33 |
| *Intercept residual* |  | 663.72 | 2.10 |  | 659.62 | 667.84 | 563.43 | 1.77 |  | 559.96 | 566.92 |

*Note*. Significant findings of interest are highlighted in bold. ICC = intraclass correlation coefficient; CI = confidence interval; AIC = Aikake information criterion; BIC = Bayesian information criterion.

For emotions, full model *ICC =* .083, *SE =* .002, 95% *CI =* .080 - .086, Log-likelihood = -1026613.8, *AIC =*2053254, *BIC =* 2053387; model with covariates: *ICC =* 1.74e-16, *SE =* 0, 95% *CI =* 1.74e-16 - 1.74e-16, Log-likelihood = -1111763.2, *AIC =* 2223550, *BIC =* 2223674; intercept-only model: *ICC =* .005, *SE =* .001, 95% *CI =* .003 - .007, Log-likelihood = -1360813.1, *AIC =* 2721632, *BIC =* 2721664.

For intentions, full model *ICC =* .147, *SE =* .002, 95% *CI =* .143 - .151, Log-likelihood = -1019073.5, *AIC =*2038173, *BIC =* 2038307; model with covariates: *ICC =* .035, *SE =* .001, 95% *CI =* .032 - .037, Log-likelihood = -1096085, *AIC =* 2192194, *BIC =* 2192318; intercept-only model: *ICC =* .043, *SE =* .001, 95% *CI =* .041 - .046, Log-likelihood = -1341257.1, *AIC =* 2682520, *BIC =* 2682552.

*Association between attachment style and touch source (partner or someone else)*

Participants chose who to hold in mind when rating imagined touch. Because person variables might have influenced participants’ selected touch source, we ran an exploratory analysis (not pre-registered) to examine whether attachment style predicted touch source. We focused on attachment style for this analysis given that touch source centred on the relational aspect of touch. We specified a bootstrapped logistic regression model (1000 replications) with attachment anxiety, attachment avoidance, and their interaction as predictors (all mean centred), and touch source (partner vs. someone else) as the outcome, as participants chose who to have in mind when rating the touch. Both higher attachment anxiety (*OR* = 1.05, *SE* = .01, *p* < .001, 95% CIs = 1.03, 1.08) and higher attachment avoidance (*OR* = 1.46, *SE* = .02, *p* < .001, 95% CIs = 1.42, 1.51) were significantly associated with higher odds of thinking of someone else, while the interaction between both dimensions was non-significant (*OR* = ,99, *SE* = .01, *p* = .183, 95% CIs = .97, 1.01). Of note, we do not know whether people with higher scores had a partner to think about, which should be considered when interpreting these results.

**Supplementary Table 5.** *Effects of person in mind (friend, partner, family member) and gender on love (emotion) and support (intention) ratings.*

|  |  | **Emotions (*N* = 9,613)** | | | | |  | **Intentions (*N* = 9,673)** | | | | |  |
| --- | --- | --- | --- | --- | --- | --- | --- | --- | --- | --- | --- | --- | --- |
|  |  | *b* | *SE* | *p* | [95% Conf.  Interval] | | Wald χ^2^ test | *b* | *SE* | *p* | [95% Conf.  Interval] | | Wald χ^2^ test |
| *Intercept* |  | 17.30 | 2.11 | < .001 | 13.17 | 21.44 |  | 29.08 | 2.87 | < .001 | 23.46 | 34.70 |  |
| *Covariates* |  |  |  |  |  |  |  |  |  |  |  |  |  |
| Last time touched  (Over a year ago = ref category) | >1 month ago | 1.02 | 2.01 | .610 | -2.91 | 4.96 |  | 2.90 | 2.73 | .288 | -2.45 | 8.25 |  |
|  | Last month or less | 2.86 | 1.88 | .127 | -0.82 | 6.54 |  | 4.99 | 2.56 | .051 | -0.03 | 10.01 |  |
|  | Last week or less | 4.89 | 1.77 | .006 | 1.42 | 8.37 |  | 5.15 | 2.43 | .034 | 0.39 | 9.91 |  |
|  | Last day or less | 6.20 | 1.75 | < .001 | 2.76 | 9.63 |  | 6.91 | 2.40 | .004 | 2.21 | 11.61 |  |
|  | Last hour | 6.76 | 1.77 | < .001 | 3.30 | 10.22 |  | 7.96 | 2.42 | .001 | 3.22 | 12.70 |  |
| Empathy Quotient |  | 0.33 | 0.04 | < .001 | 0.24 | 0.41 |  | 0.53 | 0.06 | < .001 | 0.42 | 0.65 |  |
| Attitudes to intimate touch |  | 10.00 | 0.19 | < .001 | 9.62 | 10.38 |  | 5.04 | 0.26 | < .001 | 4.54 | 5.55 |  |
| Week since start of 2020 |  | 0.05 | 0.06 | .372 | -0.06 | 0.17 |  | -0.01 | 0.08 | .902 | -0.17 | 0.15 |  |
| Interoceptive sensibility |  | 1.37 | 0.20 | < .001 | 0.98 | 1.76 |  | 1.00 | 0.26 | < .001 | 0.48 | 1.52 |  |
| *Predictor of interest* |  |  |  |  |  |  |  |  |  |  |  |  |  |
| Person in mind | Partner | 6.72 | 0.76 | **< .001** | 5.23 | 8.21 | χ^2^(2) = 79.88, p **< .001** | -0.15 | 1.01 | .879 | -2.13 | 1.82 | χ^2^(2) = 1.93, p = .380 |
|  | Stranger | 2.73 | 1.60 | .089 | -0.41 | 5.88 |  | 2.71 | 2.16 | .209 | -1.52 | 6.94 |  |
| Gender |  | -2.49 | 0.78 | **.001** | -4.02 | -0.95 |  | 1.04 | 1.04 | .316 | -0.99 | 3.07 |  |
| Person in mind x gender | Partner x female | 4.54 | 0.90 | **< .001** | 2.78 | 6.29 | χ^2^(2) = 26.62, p **< .001** | 2.39 | 1.19 | **.044** | 0.07 | 4.72 | χ^2^(2) = 4.70, p = .095 |
|  | Stranger | 4.96 | 1.76 | **.005** | 1.51 | 8.40 |  | 3.50 | 2.35 | .137 | -1.11 | 8.12 |  |
| Participant (random intercept) |  | 123.12 | 4.06 |  | 115.41 | 131.35 |  | 252.08 | 6.96 |  | 238.80 | 266.10 |  |
| Intercept residual |  | 255.76 | 3.69 |  | 248.63 | 263.10 |  | 384.18 | 5.53 |  | 373.50 | 395.17 |  |

*Note.* Significant findings of interest highlighted in bold.

*
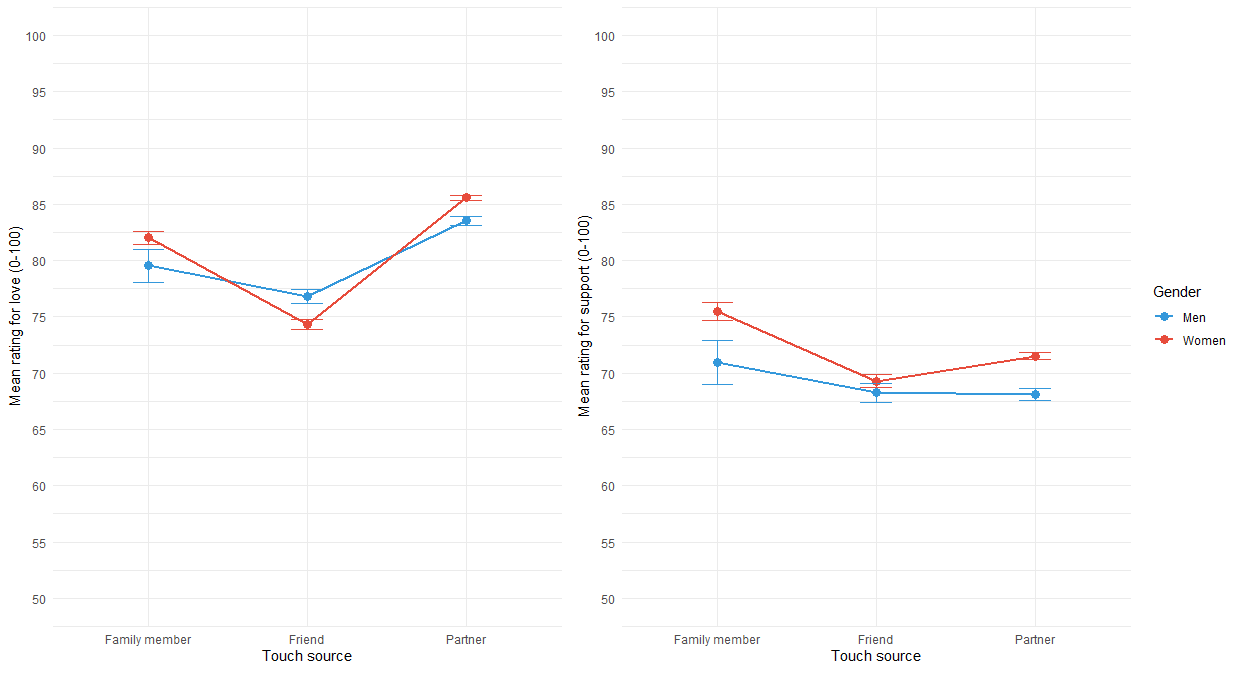
*

**Supplementary Figure 1.** *Interaction between touch source and gender for love (left panel) and support (right panel). Standard errors denote ±1 standard error of the mean.*

*Note. For love, the sample size was N = 9,613 for this analysis as we included only people who thought of their partner, family member, or friend. Of these participants, n = 879 thought of a family member, n = 1,995 thought of a friend, and n = 6,739 thought of their partner. For support, the sample size was N = 9,672; n = 885 thought of a family member, n = 2,022 thought of a friend, and n = 6,766 thought of their partner.*

**Supplementary Table 6.** *Effects of touch type, touch source, and their interaction, on ratings for emotions and intentions*.

|  |  | **Emotions (*N* = 12,501)** | | | | | **Intentions (*N* = 12,598)** | | | | |
| --- | --- | --- | --- | --- | --- | --- | --- | --- | --- | --- | --- |
|  |  | *b* | *SE* | *p* | [95% Conf. | Interval] | *b* | *SE* | *p* | [95% Conf. | Interval] |
| *Intercept* |  | 12.13 | 2.63 | < .001 | 6.98 | 17.28 | 32.97 | 2.68 | < .001 | 27.72 | 38.22 |
| *Covariates* |  |  |  |  |  |  |  |  |  |  |  |
| Last time touched | >1 month ago | -0.82 | 2.51 | .745 | -5.75 | 4.11 | 0.45 | 2.56 | .860 | -4.57 | 5.48 |
| (Over a year ago = ref category) | Last month or less | -2.70 | 2.36 | .252 | -7.31 | 1.92 | 0.07 | 2.40 | .977 | -4.63 | 4.77 |
|  | Last week or less | -2.65 | 2.21 | .231 | -6.99 | 1.69 | -0.28 | 2.26 | .902 | -4.71 | 4.15 |
|  | Last day or less | -3.16 | 2.18 | .147 | -7.44 | 1.11 | 0.56 | 2.22 | .800 | -3.80 | 4.92 |
|  | Last hour | -3.39 | 2.21 | .126 | -7.72 | 0.95 | 1.30 | 2.26 | .563 | -3.12 | 5.73 |
| Ability to empathise |  | -0.04 | 0.06 | .472 | -0.17 | 0.08 | 0.04 | 0.06 | .529 | -0.08 | 0.16 |
| Attitudes to intimate touch |  | 11.91 | 0.26 | < .001 | 11.40 | 12.42 | 7.32 | 0.26 | < .001 | 6.80 | 7.84 |
| Week since start of 2020 |  | 0.20 | 0.09 | .020 | 0.03 | 0.38 | 0.23 | 0.09 | .009 | 0.06 | 0.41 |
| Interoceptive sensibility |  | 0.39 | 0.28 | .164 | -0.16 | 0.95 | 0.12 | 0.29 | .682 | -0.44 | 0.68 |
| *Predictors of interest* |  |  |  |  |  |  |  |  |  |  |  |
| Touch type |  | -23.98 | 0.37 | **< .001** | -24.69 | -23.26 | -27.11 | 0.38 | **< .001** | -27.85 | -26.38 |
| Touch source |  | -15.97 | 0.57 | **< .001** | -17.08 | -14.86 | -16.74 | 0.57 | **< .001** | -17.87 | -15.61 |
| Touch type x touch source |  | 5.01 | 0.54 | **< .001** | 3.95 | 6.07 | 4.71 | 0.55 | **< .001** | 3.63 | 5.79 |
| Participant (random intercept) |  | 474.90 | 9.33 |  | 456.97 | 493.54 | 486.42 | 9.62 |  | 467.93 | 505.63 |
| Intercept residual |  | 453.47 | 5.74 |  | 442.36 | 464.85 | 476.71 | 6.01 |  | 465.08 | 488.63 |

*Note*. Significant findings of interest are highlighted in bold. ICC = intraclass correlation coefficient; CI = confidence interval; AIC = Aikake information criterion; BIC = Bayesian information criterion. For emotions, full model *ICC =* .512, *SE =* .007, 95% *CI =* .499 - .524, Log-likelihood = -118943.85, *AIC =* 237917.7, *BIC =* 238039.6; model with covariates: ICC = .355, *SE =* .006, 95% *CI =* .342 - .368, Log-likelihood = -177917.38, *AIC =* 355858.8, *BIC =* 355960.8; intercept-only model: *ICC =* .432, *SE =* .005, 95% *CI =* .422 - .443, Log-likelihood = -219707.36, *AIC =* 439420.7, *BIC =* 439446.8

For intentions, full model *ICC =* .505, *SE =* .007, 95% *CI =* .492 - .518, Log-likelihood = -120400.31, *AIC =* 240830.6, *BIC =* 240952.6; model with covariates: *ICC =* .303, *SE =* .007, 95% *CI =* .290 -.316, Log-likelihood = -180693.94, *AIC =* 361411.9, *BIC =* 361514; intercept-only model: *ICC =* .340, *SE =* .006, 95% *CI =* .329 - .352, Log-likelihood = -222184.06, *AIC =* 444374.1, *BIC =* 444400.3

*Relationship between childhood touch and adult attachment style*

Part of the pre-registration was to test the hypothesis that people who report less frequent touch during childhood (i.e. developmental touch history) would have less secure adult attachment. A multiple regression analysis was carried out with attachment anxiety, attachment avoidance, and their interaction as predictor variables, and childhood touch as the outcome. There was a significant negative association between attachment anxiety and developmental touch history (*b* = -.04, *SE =* .006, *p <* .001), and attachment avoidance and developmental touch history (*b* = -.23, *SE =* .007, *p <* .001), supporting the hypothesis: Higher levels of attachment anxiety and avoidance (i.e., less secure adult attachment) were related to a less positive developmental touch history. Effect size was large for attachment avoidance (partial η^2^ = .047 [95% CIs .041; .053]) and small for anxiety (partial η^2^ = .002 [95% CIs .001; .004]). The interaction between attachment anxiety and avoidance on developmental touch history was non-significant (*b* = -.003, *SE =* .005, *p =* .491).


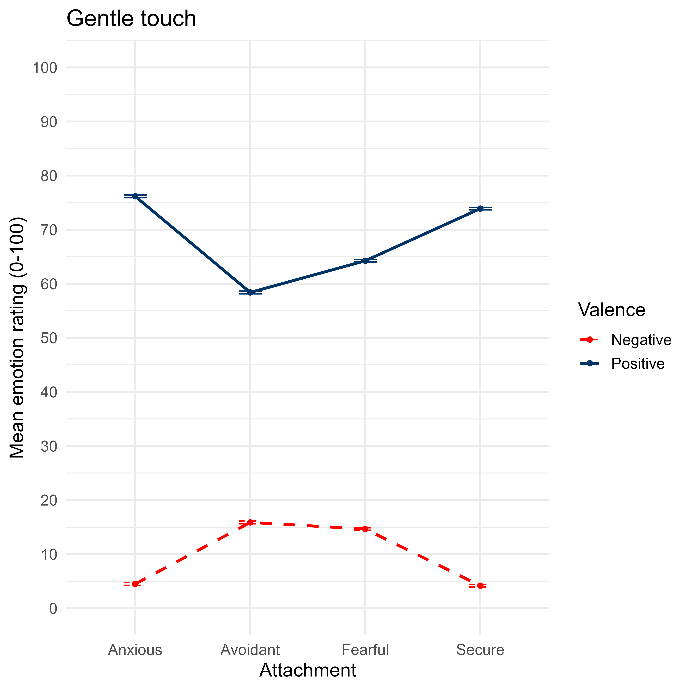

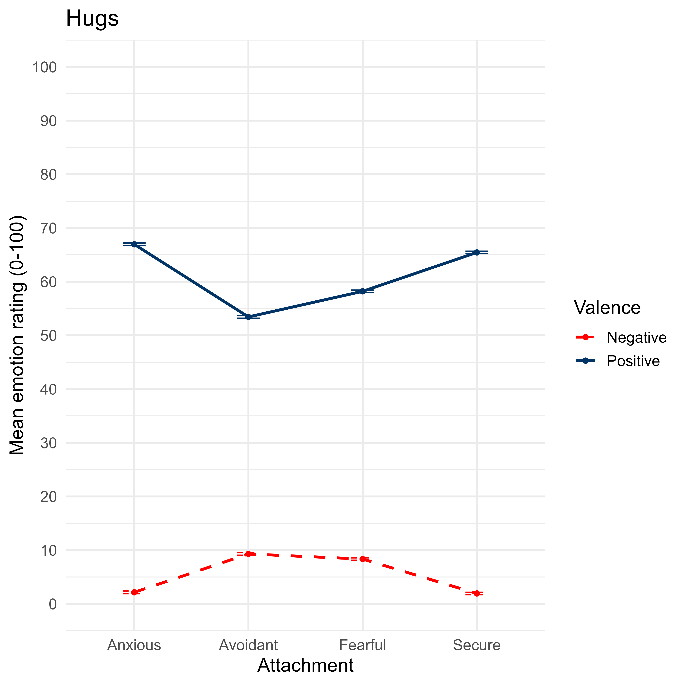


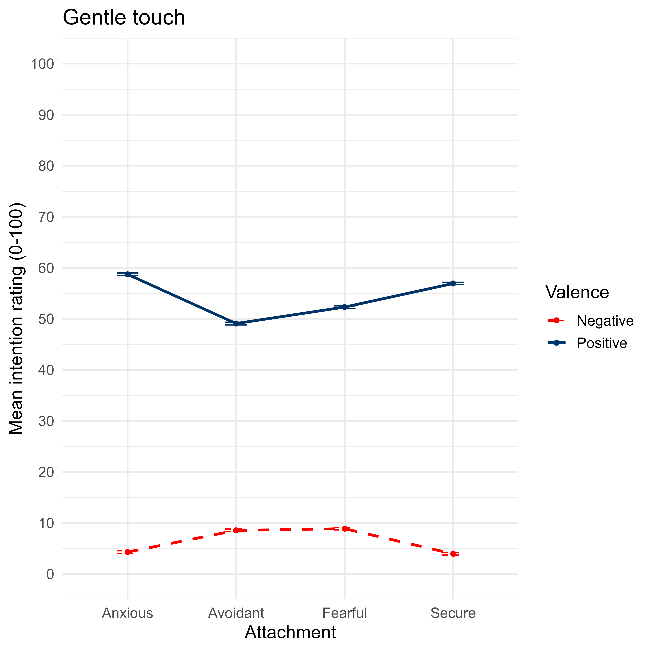

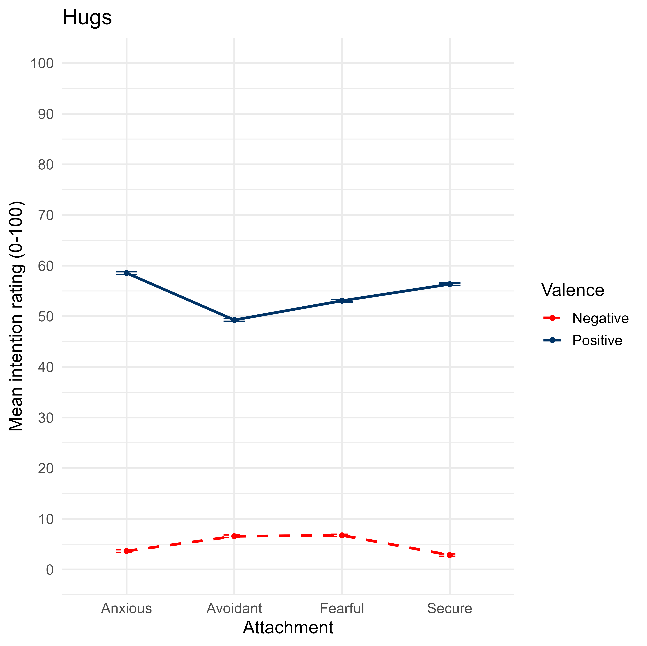


**Supplementary Figure 2.** *Valence by attachment anxiety by attachment avoidance interactions separately for gentle touch (left panels) and hugs (right panels) for emotion ratings (top panel) and intention ratings (bottom panel).* *Note that attachment styles are marginal means at +1SD anxiety/-1SD avoidance for anxious, -1SD anxiety/+1SD avoidance for avoidant, +1SD anxiety/+1SD avoidance for fearful, and -1SD anxiety/-1SD avoidance for secure attachment, meaning participants were not assigned to attachment categories. Error bars denote ± 1 standard error of the mean.*

**Supplementary Table 7**. *Planned contrasts for attachment and valence.*

|  |  | **Emotions** |  |  |  |  | **Intentions** | |  |  |  |
| --- | --- | --- | --- | --- | --- | --- | --- | --- | --- | --- | --- |
|  |  | *Contrast* | *SE* | *Bonferroni p* | *Bonferroni 95% CI* | | *Contrast* | *SE* | *Bonferroni p* | *Bonferroni 95% CI* | |
| Avoidant vs. Secure | Negative | 4.17 | 0.29 | **< .001** | 3.40 | 4.93 | 4.17 | 0.29 | **< .001** | 3.40 | 4.93 |
| Avoidant vs. Secure | Positive | -7.45 | 0.29 | **< .001** | -8.21 | -6.68 | -7.45 | 0.29 | **< .001** | -8.21 | -6.68 |
| Anxious vs. Secure | Negative | 0.60 | 0.28 | .199 | -0.14 | 1.34 | 0.60 | 0.28 | .198 | -0.14 | 1.34 |
| Anxious vs. Secure | Positive | 2.01 | 0.28 | **< .001** | 1.27 | 2.75 | 2.01 | 0.28 | **< .001** | 1.27 | 2.75 |
| Fearful vs. Secure | Negative | 4.42 | 0.29 | **< .001** | 3.66 | 5.19 | 4.42 | 0.29 | **< .001** | 3.66 | 5.19 |
| Fearful vs. Secure | Positive | -3.89 | 0.29 | **< .001** | -4.66 | -3.13 | -3.89 | 0.29 | **< .001** | -4.66 | -3.13 |

*Note*. Significant findings highlighted in bold. Secure is the reference category. Note that attachment styles are marginal means at +1SD anxiety/-1SD avoidance for anxious, -1SD anxiety/+1SD avoidance for avoidant, +1SD anxiety/+1SD avoidance for fearful, and -1SD anxiety/-1SD avoidance for secure attachment, meaning participants were not assigned to attachment categories.

**Supplementary Table 8.** *Interaction between valence, attachment anxiety, and attachment avoidance on ratings for emotions and intentions specifically for people who thought of their partner.*

|  |  | **Emotions (*N* = 6,733)** | | | | | **Intentions (*N* = 6,760)** | | | | |
| --- | --- | --- | --- | --- | --- | --- | --- | --- | --- | --- | --- |
|  |  | *b* | *SE* | *p* | [95% Conf.  Interval] | | *b* | *SE* | *p* | [95% Conf.  Interval] | |
| *Intercept* |  | -17.42 | 1.66 | **< .001** | -20.66 | -14.17 | -13.90 | 2.11 | **< .001** | -18.04 | -9.76 |
| *Covariates* |  |  |  |  |  |  |  |  |  |  |  |
| Last time touched  (Over a year ago = ref category) | >1 month ago | 1.57 | 1.54 | .308 | -1.45 | 4.59 | 2.58 | 1.97 | .191 | -1.28 | 6.44 |
|  | Last month or less | -0.97 | 1.42 | .493 | -3.76 | 1.81 | -0.37 | 1.82 | .837 | -3.94 | 3.19 |
|  | Last week or less | -1.58 | 1.32 | .234 | -4.17 | 1.02 | -0.76 | 1.70 | .653 | -4.09 | 2.56 |
|  | Last day or less | -1.76 | 1.31 | .176 | -4.32 | 0.79 | -0.37 | 1.67 | .824 | -3.65 | 2.91 |
|  | Last hour | -1.88 | 1.31 | .153 | -4.45 | 0.70 | -0.22 | 1.69 | .894 | -3.53 | 3.08 |
| Empathy Quotient |  | 0.14 | 0.03 | < .001 | 0.09 | 0.20 | 0.13 | 0.04 | < .001 | 0.06 | 0.20 |
| Attitudes to intimate touch |  | 3.80 | 0.14 | < .001 | 3.52 | 4.08 | 2.26 | 0.18 | < .001 | 1.90 | 2.61 |
| Week since start of 2020 |  | 0.03 | 0.04 | .494 | -0.05 | 0.10 | 0.07 | 0.05 | .192 | -0.03 | 0.16 |
| Interoceptive sensibility |  | 0.35 | 0.14 | .100 | 0.08 | 0.62 | 0.44 | 0.17 | .100 | 0.11 | 0.78 |
| Friend in mind bond |  | 0.38 | 0.08 | < .001 | 0.23 | 0.54 | 0.47 | 0.10 | < .001 | 0.28 | 0.66 |
| *Predictor of interest* |  |  |  |  |  |  |  |  |  |  |  |
| Valence |  | 67.05 | 0.16 | **< .001** | 66.74 | 67.36 | 54.39 | 0.16 | **< .001** | 54.07 | 54.70 |
| Attachment anxiety |  | -0.22 | 0.10 | .250 | -0.41 | -0.03 | 0.11 | 0.12 | .365 | -0.12 | 0.33 |
| Attachment avoidance |  | 2.24 | 0.13 | **< .001** | 2.00 | 2.49 | 0.88 | 0.15 | **< .001** | 0.58 | 1.17 |
| Valence x Attachment anxiety |  | 1.56 | 0.11 | **< .001** | 1.34 | 1.78 | 0.93 | 0.11 | **< .001** | 0.70 | 1.15 |
| Valence x Attachment avoidance |  | -5.75 | 0.14 | **< .001** | -6.02 | -5.48 | -3.26 | 0.14 | **< .001** | -3.54 | -2.99 |
| Attachment anxiety x Attachment avoidance |  | -0.11 | 0.08 | .150 | -0.26 | 0.04 | -0.03 | 0.09 | .766 | -0.21 | 0.15 |
| Valence x Attachment anxiety x Attachment avoidance |  | 0.66 | 0.09 | **< .001** | 0.48 | 0.84 | 0.33 | 0.09 | **< .001** | 0.15 | 0.52 |
| Participant (random intercept) |  | 35.85 | 1.31 |  | 33.37 | 38.52 | 79.76 | 2.09 |  | 75.76 | 83.98 |
| Intercept residual |  | 472.08 | 2.45 |  | 467.29 | 476.92 | 496.26 | 2.57 |  | 491.24 | 501.33 |

*Note*. Significant findings of interest highlighted in bold.

*Exploratory analyses only for people who had thought of their partner*

When we examined only participants who had thought of their partner, the anxiety by avoidance interaction was significant for both emotions (*p =* .023) and intentions (*p =* .008; see Supplementary Table 9). Plotting scores at -1*SD* and +1*SD* for anxiety and avoidance, marginal means were highest for anxious attachment scores (*M =* 49.45, *SE =* .21 for emotions; *M =* 41.82 for intentions), followed by secure (*M =* 49.16, *SE =* .18 for emotions, Bonferroni-corrected contrast anxious vs. secure was non-significant, *p =* .774; *M =* 41.38, *SE =* .23 for intentions, Bonferroni-corrected contrast anxious vs. secure was non-significant, *p =* .544), followed by fearful (*M =* 47.33, *SE =* .22, significantly lower than secure for emotions, *p <* .001; *M =* 40.19, *SE =* .23, significantly lower than secure for intentions, *p =* .001) and lastly avoidant attachment scores (*M =* 49.45, *SE =* .26, significantly lower than secure for emotions, *p <* .001; *M =* 38.45, *SE =* .30, significantly lower than secure for emotions, *p <* .001). Therefore, while avoidant/fearful attachment scores were associated with lower distinctness of emotions and intentions, anxious and secure attachment scores were associated with greater distinctness.

**Supplementary Table 9**. *Bootstrapped regression analysis for effects of attachment anxiety and attachment avoidance on distinctness of emotions and intentions for people who thought of their partner.*

|  |  | **Emotions (*N* = 6,736)** | | | | | **Intentions (*N* = 6,763)** | | | | |
| --- | --- | --- | --- | --- | --- | --- | --- | --- | --- | --- | --- |
|  |  | *b* | *SE* | *p* | [95% Conf. | Interval] | *b* | *SE* | *p* | [95% Conf. | Interval] |
| *Intercept* |  | 21.84 | 1.76 | < .001 | 18.39 | 25.30 | 22.96 | 2.24 | < .001 | 18.57 | 27.36 |
| *Covariates* |  |  |  |  |  |  |  |  |  |  |  |
| Last time touched | >1 month ago | 1.70 | 1.63 | .298 | -1.50 | 4.90 | 0.55 | 2.17 | .800 | -3.70 | 4.80 |
| (Over a year ago = ref category) | Last month or less | -1.00 | 1.57 | .526 | -4.08 | 2.09 | -0.58 | 2.10 | .782 | -4.70 | 3.54 |
|  | Last week or less | -0.50 | 1.48 | .735 | -3.41 | 2.40 | -0.18 | 2.05 | .930 | -4.19 | 3.83 |
|  | Last day or less | -0.35 | 1.47 | .813 | -3.23 | 2.53 | 0.53 | 2.00 | .792 | -3.40 | 4.45 |
|  | Last hour | -0.20 | 1.47 | .890 | -3.07 | 2.67 | 0.91 | 2.01 | .648 | -3.02 | 4.84 |
| Empathy Quotient |  | 0.17 | 0.03 | < .001 | 0.12 | 0.23 | 0.24 | 0.03 | < .001 | 0.18 | 0.30 |
| Attitudes to intimate touch |  | 4.58 | 0.17 | < .001 | 4.24 | 4.91 | 2.41 | 0.19 | < .001 | 2.04 | 2.78 |
| Week since start of 2020 |  | 0.07 | 0.04 | .061 | 0.00 | 0.14 | 0.03 | 0.05 | .551 | -0.06 | 0.12 |
| Interoceptive sensibility |  | 0.73 | 0.13 | < .001 | 0.48 | 0.98 | 0.68 | 0.15 | < .001 | 0.37 | 0.98 |
| *Predictor of interest* |  |  |  |  |  |  |  |  |  |  |  |
| Attachment anxiety |  | 0.32 | 0.08 | **< .001** | 0.16 | 0.47 | 0.44 | 0.09 | **< .001** | 0.25 | 0.62 |
| Attachment avoidance |  | -1.14 | 0.10 | **< .001** | -1.34 | -0.93 | -0.99 | 0.12 | **< .001** | -1.23 | -0.75 |
| Attachment anxiety x Attachment avoidance |  | 0.15 | 0.07 | **.023** | 0.02 | 0.29 | 0.20 | 0.08 | **.008** | 0.05 | 0.35 |

*Note*. Significant findings of interest highlighted in bold.

**Supplementary Table 10.** *Interaction between valence and positive childhood touch on ratings for emotions and intentions.*

|  |  | **Emotions (*N* = 18,218)** | | | | | **Intentions (*N* = 18,336)** | | | | |
| --- | --- | --- | --- | --- | --- | --- | --- | --- | --- | --- | --- |
|  |  | *b* | *SE* | *p* | [95% Conf. | Interval] | *b* | *SE* | *p* | [95% Conf. | Interval] |
| *Intercept* |  | -7.78 | 0.89 | < .001 | -9.52 | -6.04 | -6.10 | 0.99 | < .001 | -8.04 | -4.16 |
| *Covariates* |  |  |  |  |  |  |  |  |  |  |  |
| Last time touched | >1 month ago | -0.63 | 0.87 | .471 | -2.33 | 1.08 | -0.54 | 0.98 | .576 | -2.46 | 1.37 |
| (Over a year ago = ref category) | Last month or less | -1.29 | 0.81 | .111 | -2.88 | 0.30 | -1.41 | 0.91 | .121 | -3.19 | 0.37 |
|  | Last week or less | -0.81 | 0.76 | .289 | -2.31 | 0.69 | -1.00 | 0.86 | .243 | -2.68 | 0.68 |
|  | Last day or less | -0.11 | 0.75 | .885 | -1.58 | 1.37 | -0.35 | 0.85 | .676 | -2.01 | 1.31 |
|  | Last hour | 0.45 | 0.76 | .559 | -1.05 | 1.94 | 0.08 | 0.86 | .928 | -1.61 | 1.76 |
| Ability to empathise |  | 0.24 | 0.02 | < .001 | 0.19 | 0.28 | 0.25 | 0.02 | < .001 | 0.20 | 0.29 |
| Attitudes to intimate touch |  | 4.09 | 0.09 | < .001 | 3.92 | 4.26 | 2.53 | 0.10 | < .001 | 2.34 | 2.72 |
| Week since start of 2020 |  | 0.05 | 0.03 | .128 | -0.01 | 0.11 | 0.05 | 0.03 | .160 | -0.02 | 0.11 |
| Interoceptive sensibility |  | 0.11 | 0.09 | .231 | -0.07 | 0.30 | 0.22 | 0.11 | .035 | 0.02 | 0.43 |
| *Predictors of interest* |  |  |  |  |  |  |  |  |  |  |  |
| Valence |  | 44.80 | 0.29 | **< .001** | 44.22 | 45.37 | 41.66 | 0.27 | **< .001** | 41.13 | 42.20 |
| Positive childhood touch |  | -1.67 | 0.07 | **< .001** | -1.82 | -1.53 | -0.85 | 0.08 | **< .001** | -1.00 | -0.70 |
| Valence x Positive childhood touch |  | 3.72 | 0.08 | **< .001** | 3.55 | 3.88 | 2.12 | 0.08 | **< .001** | 1.97 | 2.27 |
| Participant (random intercept) |  | 60.80 | 1.22 |  | 58.44 | 63.24 | 97.44 | 1.51 |  | 94.52 | 100.46 |
| Intercept residual |  | 657.04 | 2.08 |  | 652.98 | 661.12 | 561.24 | 1.77 |  | 557.79 | 564.72 |

*Note*. Significant findings of interest highlighted in bold. ICC = intraclass correlation coefficient; CI = confidence interval; AIC = Aikake information criterion; BIC = Bayesian information criterion. For emotions, full model *ICC =* .085, SE = .002, 95% *CI =* .082 - .088, Log-likelihood = -1025263.4, *AIC =* 2050557, *BIC =* 2050711; model with covariates: ICC = 1.75e-16, *SE =* 0, 95% *CI =* 1.75e-16 - 1.75e-16, Log-likelihood = -1111763.2, *AIC =* 2223550, *BIC =* 2223674; intercept-only model: *ICC =* .005, SE = .001, 95% *CI =* .003 - .007, Log-likelihood = -1360813.1, *AIC =* 2721632, *BIC =* 2721664. For intentions, full model *ICC =* .148, *SE =* .002, 95% *CI =* .143 - .152, Log-likelihood = -1018288.9, *AIC =* 2036608, *BIC =* 2036762; model with covariates: ICC = 1.75e-16, *SE =* 0, 95% *CI =* 1.75e-16 - 1.75e-16, Log-likelihood = -1111763.2, *AIC =* 2223550, *BIC =* 2223674; intercept-only model: *ICC =* .005, SE = .001, 95% *CI =* .003 - .007, Log-likelihood = -1360813.1, *AIC =* 2721632, *BIC =* 2721664.


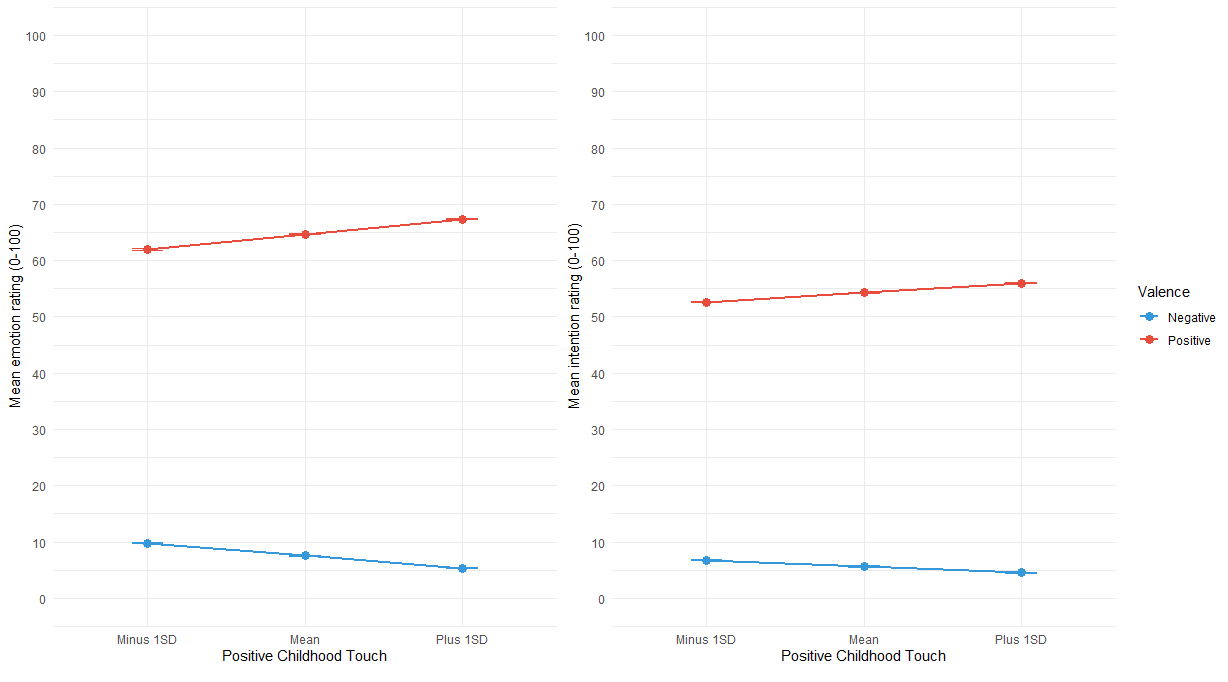


**Supplementary Figure 3.** *Valence by positive childhood positive touch interaction for emotions (left panel) and intentions (right panel). Positive childhood touch was a continuous variable, which we plotted at -1SD, mean, and +1SD values. Error bars denote ±1 standard error of the mean.***Supplementary Table 11.** *Bootstrapped regression analysis for effects of positive childhood touch on distinctness of emotions and intentions.*

|  |  | **Emotions (*N* = 18,218)** | | | | | **Intentions (*N* = 18,336)** | | | | |
| --- | --- | --- | --- | --- | --- | --- | --- | --- | --- | --- | --- |
|  |  | *b* | *SE* | *p* | [95% Conf. | Interval] | *b* | *SE* | *p* | [95% Conf. | Interval] |
| *Intercept* |  | 12.47 | 1.16 | < .001 | 10.20 | 14.74 | 13.38 | 1.09 | < .001 | 11.24 | 15.51 |
| *Covariates* |  |  |  |  |  |  |  |  |  |  |  |
| Last time touched | >1 month ago | -0.86 | 1.16 | .461 | -3.13 | 1.42 | 0.81 | 1.07 | .450 | -1.29 | 2.91 |
| (Over a year ago = ref category) | Last month or less | -1.20 | 1.09 | .273 | -3.34 | 0.94 | 1.15 | 1.01 | .258 | -0.84 | 3.14 |
|  | Last week or less | 0.12 | 1.05 | .909 | -1.93 | 2.17 | 1.88 | 0.97 | .052 | -0.02 | 3.78 |
|  | Last day or less | 0.82 | 1.04 | .431 | -1.22 | 2.87 | 2.69 | 0.97 | .005 | 0.80 | 4.59 |
|  | Last hour | 1.69 | 1.04 | .105 | -0.35 | 3.74 | 3.23 | 0.97 | .001 | 1.34 | 5.13 |
| Ability to empathise |  | 0.29 | 0.02 | < .001 | 0.25 | 0.33 | 0.35 | 0.02 | < .001 | 0.31 | 0.39 |
| Attitudes to intimate touch |  | 4.69 | 0.10 | < .001 | 4.50 | 4.89 | 2.96 | 0.10 | < .001 | 2.77 | 3.15 |
| Week since start of 2020 |  | 0.30 | 0.03 | < .001 | 0.24 | 0.35 | 0.16 | 0.03 | < .001 | 0.10 | 0.22 |
| Interoceptive sensibility |  | 0.84 | 0.09 | < .001 | 0.65 | 1.02 | 0.61 | 0.10 | < .001 | 0.43 | 0.80 |
| *Predictors of interest* |  |  |  |  |  |  |  |  |  |  |  |
| Positive childhood touch |  | 0.52 | 0.06 | **< .001** | 0.40 | 0.63 | 0.46 | 0.06 | **< .001** | 0.34 | 0.58 |

*Note*. Significant findings of interest highlighted in bold.
